# Supplementary material for: CAMITAX: Taxon labels for microbial genomes
Source: Gigascience. 2020 Jan 7;9(1):giz154. doi: 10.1093/gigascience/giz154 (PMC6946028; doi:10.1093/gigascience/giz154)
Supplement: giz154_GIGA-D-19-00212_Original_Submission [file giz154_giga-d-19-00212_original_submission.pdf]

# GigaScience


# CAMITAX: Taxon labels for microbial genomes

--Manuscript Draft--

| | |
|---|---|
| **Manuscript Number:** | GIGA-D-19-00212 |
| **Full Title:** | CAMITAX: Taxon labels for microbial genomes |
| **Article Type:** | Technical Note |
| **Funding Information:** | |
| **Abstract:** | The number of microbial genome sequences is growing exponentially, also thanks to recent advances in recovering complete or near-complete genomes from metagenomes and single cells. Assigning reliable taxon labels to genomes is key and often a prerequisite for downstream analyses. We introduce CAMITAX, a scalable and reproducible workflow for the taxonomic labelling of microbial genomes recovered from isolates, single cells, and metagenomes. CAMITAX combines genome distance-, 16S rRNA gene-, and gene homology-based taxonomic assignments with phylogenetic placement. It uses Nextflow to orchestrate reference databases and software containers, and thus combines ease of installation and use with computational reproducibility. We evaluated the method on several hundred metagenome-assembled genomes with high-quality taxonomic annotations from the TARA Oceans project, and show that the ensemble classification method in CAMITAX improved on all individual methods across tested ranks. While we initially developed CAMITAX to aid the Critical Assessment of Metagenome Interpretation (CAMI) initiative, it evolved into a comprehensive software to reliably assign taxon labels to microbial genomes. CAMITAX is available under the Apache License 2.0 at: https://github.com/CAMI-challenge/CAMITAX |
| **Corresponding Author:** | Andreas Bremges |
| **Corresponding Author Secondary Information:** | |
| **Corresponding Author's Institution:** | |
| **Corresponding Author's Secondary Institution:** | |
| **First Author:** | Andreas Bremges |
| **First Author Secondary Information:** | |
| **Order of Authors:** | Andreas Bremges |
| | Adrian Fritz |
| | Alice C McHardy |
| **Order of Authors Secondary Information:** | |
| **Additional Information:** | |
| **Question** | **Response** |
| Are you submitting this manuscript to a special series or article collection? | No |
| **Experimental design and statistics**<br><br>Full details of the experimental design and statistical methods used should be given in the Methods section, as detailed in our Minimum Standards Reporting Checklist. | Yes |

Powered by Editorial Manager® and ProduXion Manager® from Aries Systems Corporation

| | |
|---|---|
| Information essential to interpreting the data presented should be made available in the figure legends.<br><br>Have you included all the information requested in your manuscript? | |
| **Resources**<br><br>A description of all resources used, including antibodies, cell lines, animals and software tools, with enough information to allow them to be uniquely identified, should be included in the Methods section. Authors are strongly encouraged to cite Research Resource Identifiers (RRIDs) for antibodies, model organisms and tools, where possible.<br><br>Have you included the information requested as detailed in our Minimum Standards Reporting Checklist? | Yes |
| **Availability of data and materials**<br><br>All datasets and code on which the conclusions of the paper rely must be either included in your submission or deposited in publicly available repositories (where available and ethically appropriate), referencing such data using a unique identifier in the references and in the "Availability of Data and Materials" section of your manuscript.<br><br>Have you have met the above requirement as detailed in our Minimum Standards Reporting Checklist? | Yes |

Powered by Editorial Manager® and ProduXion Manager® from Aries Systems Corporation

Click here to download Manuscript camitax.pdf



# CAMITAX: Taxon labels for microbial genomes

**Andreas Bremges**[1,2,*], **Adrian Fritz**[1], and **Alice C. McHardy**[1,*]

[1] Computational Biology of Infection Research, Helmholtz Centre for Infection Research, 38124 Braunschweig, Germany
[2] German Center for Infection Research (DZIF), partner site Hannover-Braunschweig, 38124 Braunschweig, Germany

[*] Correspondence: andreas.bremges@helmholtz-hzi.de; alice.mchardy@helmholtz-hzi.de

**The number of microbial genome sequences is growing exponentially, also thanks to recent advances in recovering complete or near-complete genomes from metagenomes and single cells. Assigning reliable taxon labels to genomes is key and often a prerequisite for downstream analyses. We introduce CAMITAX, a scalable and reproducible workflow for the taxonomic labelling of microbial genomes recovered from isolates, single cells, and metagenomes. CAMITAX combines genome distance-, 16S rRNA gene-, and gene homology-based taxonomic assignments with phylogenetic placement. It uses Nextflow to orchestrate reference databases and software containers, and thus combines ease of installation and use with computational reproducibility. We evaluated the method on several hundred metagenome-assembled genomes with high-quality taxonomic annotations from the TARA Oceans project, and show that the ensemble classification method in CAMITAX improved on all individual methods across tested ranks. While we initially developed CAMITAX to aid the Critical Assessment of Metagenome Interpretation (CAMI) initiative, it evolved into a comprehensive software to reliably assign taxon labels to microbial genomes. CAMITAX is available under the Apache License 2.0 at: https://github.com/CAMI-challenge/CAMITAX**

## INTRODUCTION

The direct costs for sequencing a microbial genome are at an all-time low: a high-quality draft now costs less than $100, a "finished" genome sequence less than $500. This resulted in many culture-dependent genome studies, in which thousands of isolates—selected by e.g. their distinct phylogeny [1, 2], abundance in the human microbiome [3, 4], or biotechnological relevance [5, 6]—are sequenced.

Single cell genome and shotgun metagenome studies further contribute to this expansion in genome numbers by enabling access to the genome sequences of (as-yet) uncultured microbes [7–9]. Notably, new bioinformatics methods can reconstruct complete or near-complete genomes even from complex environments [10, 11], and easily scale to hundreds or even thousands of metagenome samples [12–16].

Typically, the sequencing and assembly of a new genome is merely a prerequisite for further bioinformatics analyses (and their experimental validation) to uncover novel biological insights by e.g. functional annotation [17, 18] or phenotype prediction [19, 20], which often require the genome's taxonomy.

Historically, a bacterial or archaeal species was defined as a collection of strains that share one (or more) trait(s) and show DNA-DNA reassociation values of 70% or higher [21]. However, with the advent of genomics and—more recently—culture-independent methods, this definition was found to be impractical and difficult to implement [22].

Today, 16S rRNA gene similarity, average nucleotide identity (ANI), genome phylogeny, or gene-centric voting schemes are used for taxonomic assignments [23–28]. These approaches all have their merits (see below), but, to the best of our knowledge, no unifying workflow implementation existed. To jointly use these complementary approaches, we developed CAMITAX, a scalable and reproducible workflow that combines genome distance-, 16S rRNA gene-, and gene homology-based taxonomic assignments with phylogenetic placement onto a fixed reference tree to reliably infer genome taxonomy.

## METHODS

In the following, we describe CAMITAX's assignment strategies and its implementation (Figure 1).

### Genome distance-based assignment

An ANI value of 95% roughly corresponds to a 70% DNA-DNA reassociation value (the historical species definition) [24]. In other words, strains from the same species are expected to show >95% ANI [29]. This species boundary appears to be widely applicable and has been confirmed in a recent large-scale study, in which the analyses of 8 billion genome pairs revealed a clear genetic discontinuity among known genomes, with 99.8% of the pairs showing either >95% intra-species ANI or <83% inter-species ANI values [30].

CAMITAX uses Mash [31] to rapidly estimate the input genomes' ANI to all bacterial or archaeal genomes in the RefSeq database [32] (114,176 strains as of 2018-05-10). CAMITAX's genome distance-based assignment is the lowest common ancestor (LCA) of all Mash hits with >95% ANI; a genome is placed at *root* if there is no RefSeq genome with >95% ANI.

This strategy works best if the query genome is more than 80% complete (Mash does not accurately estimate the genome-wide ANI of incomplete genomes [33]) and is represented in RefSeq. CAMITAX's other assignment strategies are complementary by design and better suited for incomplete genomes or underrepresented lineages. If a Mash hit is found, however, CAMITAX most likely assigns a taxonomy at the species or genus level.

### 16S rRNA gene-based assignment

The 16S rRNA gene is widely used for classification tasks because it is an universal marker gene likely present in all bacteria and archaea [34, 35].

CAMITAX uses nhmmer [36] to identify 16S rRNA genes in the input genomes and Dada2 [37] to assign taxonomy. Dada2 uses the naïve Bayesian classifier method [38] for kingdom to genus assignments, and exact sequence matching against a reference database for species assignments. CAMITAX supports two commonly used databases: SILVA [39] and RDP [40], which both were found to map back well to the NCBI Taxonomy [41].

Of course, this strategy only is applicable if the genome assembly contains a copy of the 16S rRNA gene—which is not always the case, particularly for genomes recovered from metagenomes or single cells.

### Gene homology-based assignments

Metagenomics and single cell genomics are complementary approaches providing access to the genomes of (as-yet) uncultured microbes, but both have strings attached: Single amplified genomes (SAGs) suffer from amplification bias and, as a consequence, are often incomplete [42, 43]. Metagenome-assembled genomes (MAGs) on the other hand rarely contain full-length 16S rRNA genes [44, 45]. While there are notable exceptions to this rule [46, 47], the above assignment strategies are generally not expected to work well for today's SAGs and MAGs.

To overcome these problems, CAMITAX implements a gene-based voting scheme. It uses Prodigal [48] to predict protein-coding genes, and then Centrifuge [49] and Kaiju [50] for gene homology searches on the nucleotide and protein level, respectively. Both tools scale to large reference databases, such as NCBI's nr/nt [51], but (by default) CAMITAX resorts to the (much smaller) proGenomes genes and proteins datasets [52, 53]. The proGenomes database was designed as a resource for consistent taxonomic annotations of bacteria and archaea.

Inferring genome taxonomy from a set of gene-level assignments is not trivial, and—inspired by procedures implemented in anvi'o [27] and dRep [33]—CAMITAX places the query genome on the lowest taxonomic node with at least 50% support in gene assignments (which corresponds to the interval-union LCA algorithm [28]) for nucleotide and protein searches.

### Phylogenetic placement

CAMITAX uses CheckM [25] for a phylogeny-driven estimate of taxonomy. Relying on 43 phylogenetically informative marker genes (consisting primarily of ribosomal proteins and RNA polymerase domains), CheckM places the query genome onto a fixed reference tree with Pplacer [54] to infer taxonomy. We note that phylogenetic placement is often quite conservative and does not necessarily provide resolution at the species level [26, 55].

Lastly, CAMITAX reports the query genome's completeness and contamination as estimated by CheckM using its lineage-specific marker genes [25].

### Classification algorithm

CAMITAX considers the lowest consistent assignment as the longest unambiguous root-to-node path in the taxonomic tree spanned by the individual assignments, i.e. it retains the most specific, yet consistent taxonomic label among all tools. For example, CAMITAX would determine as "consistent" assignments for the individual assignments (derived with the different assignment strategies) the following:

- 3× *E. coli*, 2× *Bacteria* $\mapsto$ *E. coli*
- 3× *E. coli*, 2× *E. albertii* $\mapsto$ *Escherichia*
- 3× *E. coli*, 2× *Archaea* $\mapsto$ Root

This strategy is more robust than computing the lowest common ancestor (LCA) of individual assignments because outliers, e.g. missing predictions of conservative methods, don't affect the overall assignment.

At the same time, requiring a consistent assignment is less error-prone than e.g. selecting the maximal root-to-leaf path, which would introduce many false-positive assignments especially on lower ranks.

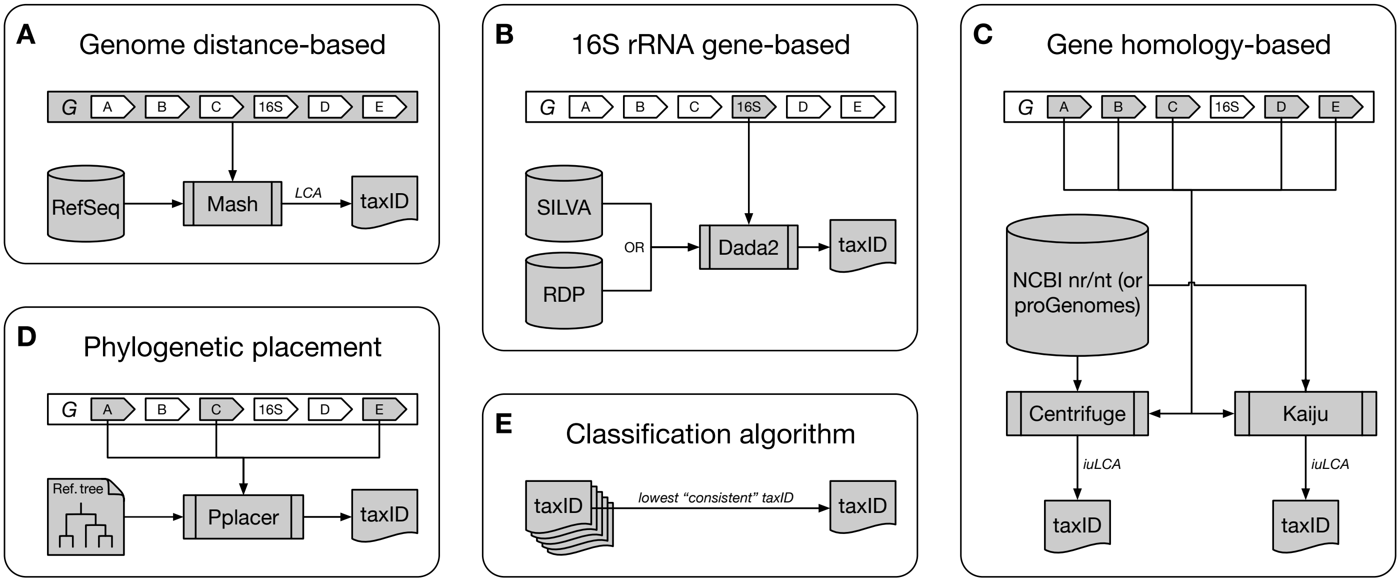


**Fig. 1. The CAMITAX taxonomic assignment workflow.** CAMITAX assigns one NCBI Taxonomy ID (taxID) to an input genome *G* by combining genome distance-, 16S rRNA gene-, and gene homology-based taxonomic assignments with phylogenetic placement. **(A) Genome distance-based assignment.** CAMITAX uses Mash to estimate the average nucleotide identity (ANI) between *G* and more than a hundred thousand microbial genomes in RefSeq, and assigns the lowest common ancestor (LCA) of genomes showing >95% ANI, which was found to be a clear species boundary. **(B) 16S rRNA gene-based assignment.** CAMITAX uses Dada2 to label *G*'s 16S rRNA gene sequences using the naïve Bayesian classifier method to assign taxonomy across multiple ranks (down to genus level), and exact sequence matching for species-level assignments, against the SILVA or RDP database. **(C) Gene homology-based assignments.** CAMITAX uses Centrifuge and Kaiju to perform gene homology searches against nucleotide and amino acid sequences in NCBI's nr and nt (or proGenomes' genes and proteins datasets), respectively. CAMITAX determines the interval-union LCA (iuLCA) of gene-level assignments and places *G* on the lowest taxonomic node with at least 50% coverage. **(D) Phylogenetic placement.** CAMITAX uses Pplacer to place *G* onto a fixed reference tree, as implemented in CheckM, and estimates genome completeness and contamination using lineage-specific marker genes. **(E) Classification algorithm.** CAMITAX considers the lowest consistent assignment as the longest unambiguous root-to-node path in the taxonomic tree spanned by the five taxIDs derived in (A)–(D), i.e. it retains the most specific, yet consistent taxonomic label among all tools.

### Implementation

CAMITAX incorporates many state-of-the-art pieces of software, and automatically resolves all software and database dependencies with Nextflow [56] in a containerized environment (Table 1). This fosters reproducibility in bioinformatics research [57, 58], and we strongly suggest to run CAMITAX using BioContainers [59] (automated container builds for software in bioconda [60]). CAMITAX can be run on a local machine or in a distributed fashion.

## RESULTS

We applied CAMITAX to real data not present in its databases, a recent collection of 885 bacterial and archaeal MAGs from Delmont *et al.* [15], who used state-of-the-art metagenomic assembly, binning, and curation strategies to create a non-redundant database of microbial population genomes from the TARA Oceans project [61].

Delmont *et al.* used CheckM for an initial taxonomic inference of the MAGs. Thereafter, they used Centrifuge [49], RAST [62], and manual BLAST searches of single-copy core genes against NCBI's nr/nt to manually refine their taxonomic inferences. Lastly, they trained a novel machine learning classifier to also identify MAGs affiliated to the Candidate Phyla Radiation (CPR) [8].

As expected, CAMITAX outperformed CheckM, which is rather conservative in its assignments, by adding low-ranking annotations based on high-quality predictions of other tools, such as Kaiju (Figure 2). Notably, 95% of CAMITAX's predictions were consistent with Delmont *et al.*, i.e. the two assignments were on the same taxonomic lineage and their LCA is either of the two. CAMITAX assignments of 46 MAGs (5%) were in conflict with the manually curated taxonomy. Of these, CAMITAX made species assignments for twelve MAGs based on Mash hits against RefSeq genomes. These we consider trustworthy because >95% ANI was shown to be a clear species boundary [30], and we assume that Delmont *et al.* assigned them incorrectly. On the other hand, CAMITAX for instance misclassified MAGs affiliated to

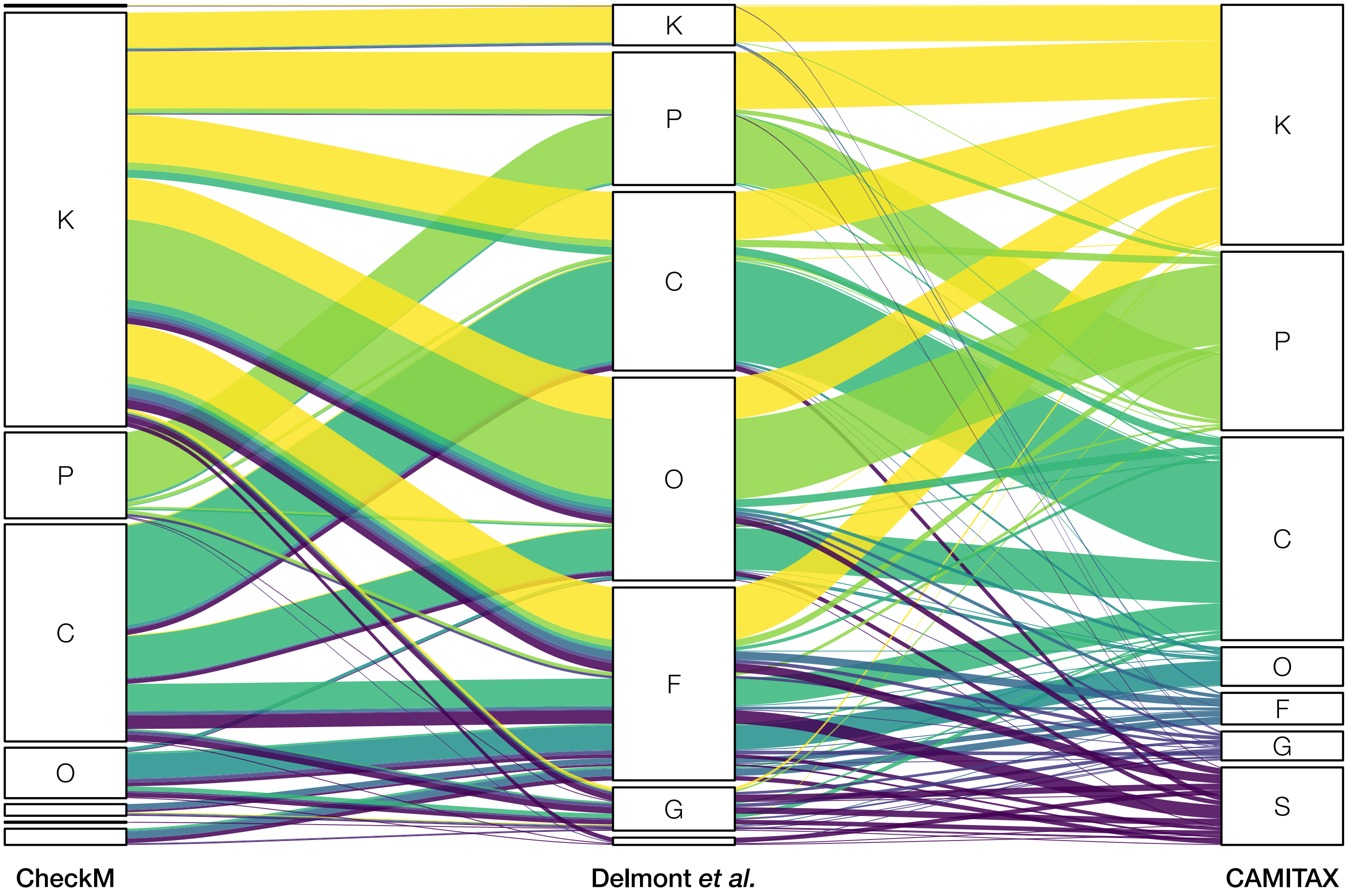


**Fig. 2. Comparison of high quality taxonomic assignments for 885 MAGs.** Using genome-resolved metagenomics, Delmont *et al.* assembled 885 bacterial and archaeal genomes from the TARA Oceans metagenomes and used CheckM for an initial taxonomic inference. Subsequently, they manually refined the taxonomic assignments using additional analyses and expert knowledge. The alluvial diagram shows the assigned taxonomic ranks for CheckM (left), manual curation (middle), and CAMITAX (right) on kingdom, phylum, class, order, family, genus, and species level.

the Candidate Phyla Radiation based on their 16S rRNA gene sequences to other phyla.

To quantify taxonomic assignment performance, we calculated precision, recall, and accuracy across all ranks with AMBER 2.0 [63] (Figure 3). As the gold standard, we used the Delmont *et al.* assignments up to genus rank. CAMITAX was very precise down to class level and reasonably (>80%) precise below. Overall, it was more accurate across all ranks than each of its assignment strategies individually. While the recall of CAMITAX dropped at the mid-range ranks, it recovered for genus level assignments.

We thus propose CAMITAX as a reliable and reproducible taxonomic assignment workflow, ideally followed by a manual refinement step—as always.

## DISCUSSION

CAMITAX was initially developed while preparing the second Critical Assessment of Metagenome Interpretation (CAMI) challenge [64]. The challenge datasets include new genomes from taxa (at different evolutionary distances) not found in public databases yet, which need high quality taxon labels for the subsequent microbial community and metagenome data simulation [65]. Due to this need, we created CAMITAX to systematically double-check, newly infer, or refine genome taxon label assignments in a fully reproducible way.

CAMITAX combines different taxonomic assignment strategies in one unifying workflow implementation. It uses Nextflow to orchestrate reference databases and software containers. Therefore, both databases and software can be easily substituted, providing the flexibility to cope with rapid change of standards oftentimes observed in the field. For instance, Parks *et al.* recently proposed a standardized bacterial taxonomy based on genome phylogeny, the so-called Genome Taxonomy Database (GTDB) [66]. While CAMITAX currently uses the NCBI Taxonomy [67], it is (at least in principle) agnostic to the underlying database and could thus be easily adapted to other taxonomy versions that will arise in future.

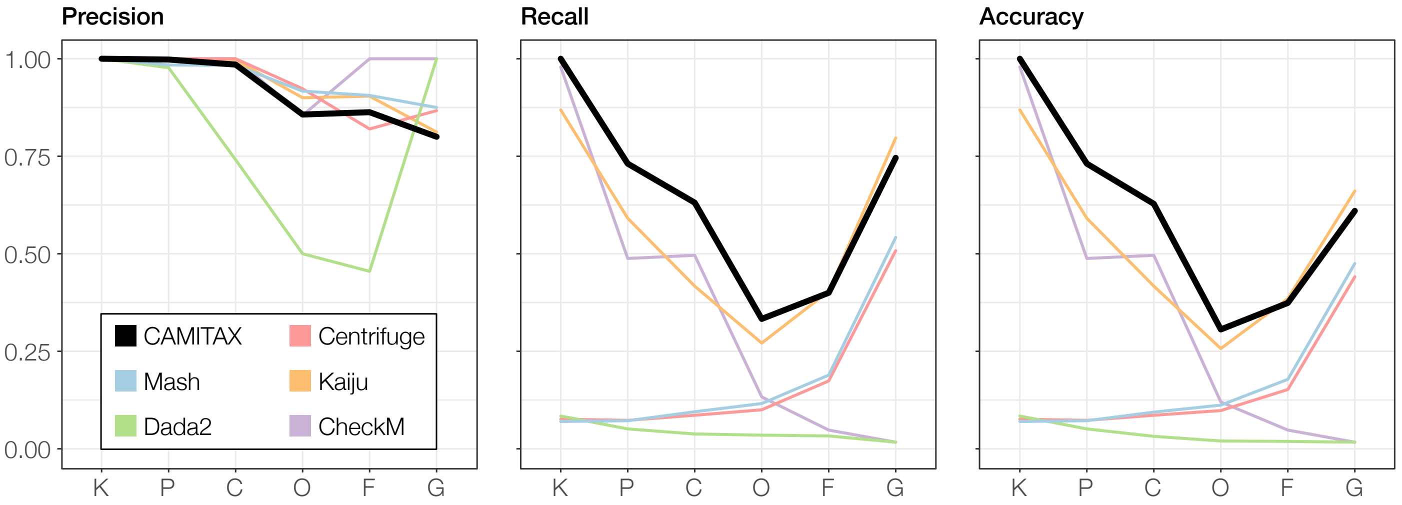


**Fig. 3. Taxonomic assignment performance metrics across ranks for 885 MAGs.** The manually assigned taxonomy by Delmont *et al.* is used as the gold standard to benchmark against. Shown are precision, recall, and accuracy for CAMITAX (and the individual tools combined therein) on kingdom, phylum, class, order, family, and genus level.

**Table 1.** Software used in the CAMITAX workflow.

| Software | Version | BioContainer |
|---|---|---|
| Centrifuge | 1.0.3 | centrifuge:1.0.3–py36pl5.22.0_2 |
| CheckM | 1.0.11 | checkm-genome:1.0.11–0 |
| Dada2 | 1.6.0 | bioconductor-dada2:1.6.0–r3.4.1_0 |
| Kaiju | 1.6.2 | kaiju:1.6.2–pl5.22.0_0 |
| Mash | 2.0 | mash:2.0–gsl2.2_2 |
| Nhmmer | 3.1 | – |
| Pplacer | 1.1 | – |
| Prodigal | 2.6.3 | prodigal:2.6.3–0 |

CAMITAX automatically resolves all software dependencies with Nextflow using BioContainers in a containerized environment. Nhmmer and Pplacer are bundled with CheckM.

## SOFTWARE AND DATA AVAILABILITY

CAMITAX is implemented in Nextflow and Python 3, and is freely available under the Apache License 2.0 at https://github.com/CAMI-challenge/CAMITAX.

Mash sketches for all bacterial and archaeal genomes in RefSeq, snapshots of the NCBI Taxonomy databases, and Centrifuge and Kaiju indices for the proGenomes genes and proteins datasets, respectively, are collected under doi:10.5281/zenodo.1250043. The snapshots used in this study, generated on 2018-05-10, are available under doi:10.5281/zenodo.1250044.

Dada2-formatted training fasta files, derived from SILVA (release 132) and RDP (training set 16, release 11.5), are available under doi:10.5281/zenodo.1172782 and doi:10.5281/zenodo.801827, respectively.

Lastly, the CheckM reference databases are available at https://data.ace.uq.edu.au/public/CheckM_databases.

## AUTHORS' CONTRIBUTIONS

AB implemented the software, performed experiments, and wrote the paper with comments from AF and ACM. AF thoroughly tested the software. AB and ACM jointly conceived the project and evaluated results. All authors read and approved the final manuscript.

## ACKNOWLEDGEMENTS

The authors thank Peter Belmann for Nextflow and Docker tips, Fernando Meyer for early beta testing, and the Isaac Newton Institute for Mathematical Sciences for its hospitality during the programme MTG, which was supported by EPSRC Grant Number EP/K032208/1.

## REFERENCES


1. D. Wu, P. Hugenholtz, K. Mavromatis, R. Pukall, E. Dalin, N. N. Ivanova, V. Kunin, L. Goodwin, M. Wu, B. J. Tindall *et al.*, "A phylogeny-driven genomic encyclopaedia of Bacteria and Archaea," Nature **462**, 1056–1060 (2009). doi:10.1038/nature08656.
2. S. Mukherjee, R. Seshadri, N. J. Varghese, E. A. Eloe-Fadrosh, J. P. Meier-Kolthoff, M. Göker, R. C. Coates, M. Hadjithomas, G. A. Pavlopoulos, D. Paez-Espino *et al.*, "1,003 reference genomes of bacterial and archaeal isolates expand coverage of the tree of life," Nat. Biotechnol. **35**, 676–683 (2017). doi:10.1038/nbt.3886.
3. H. P. Browne, S. C. Forster, B. O. Anonye, N. Kumar, B. A. Neville, M. D. Stares, D. Goulding, and T. D. Lawley, "Culturing of 'unculturable' human microbiota reveals novel taxa and extensive sporulation," Nature. **533**, 543–546 (2016). doi:10.1038/nature17645.
4. J. C. Lagier, S. Khelaifia, M. T. Alou, S. Ndongo, N. Dione, P. Hugon, A. Caputo, F. Cadoret, S. I. Traore, E. H. Seck *et al.*, "Culture of previously uncultured members of the human gut microbiota by culturomics," Nat Microbiol **1**, 16203 (2016). doi:10.1038/nmicrobiol.2016.203.

5. I. Maus, A. Bremges, Y. Stolze, S. Hahnke, K. G. Cibis, D. E. Koeck, Y. S. Kim, J. Kreubel, J. Hassa, D. Wibberg *et al.*, "Genomics and prevalence of bacterial and archaeal isolates from biogas-producing microbiomes," Biotechnol Biofuels **10**, 264 (2017). doi:10.1186/s13068-017-0947-1.
6. R. Seshadri, S. C. Leahy, G. T. Attwood, K. H. Teh, S. C. Lambie, A. L. Cookson, E. A. Eloe-Fadrosh, G. A. Pavlopoulos, M. Hadjithomas, N. J. Varghese *et al.*, "Cultivation and sequencing of rumen microbiome members from the Hungate1000 Collection," Nat. Biotechnol. **36**, 359–367 (2018). doi:10.1038/nbt.4110.
7. C. Rinke, P. Schwientek, A. Sczyrba, N. N. Ivanova, I. J. Anderson, J. F. Cheng, A. Darling, S. Malfatti, B. K. Swan, E. A. Gies *et al.*, "Insights into the phylogeny and coding potential of microbial dark matter," Nature. **499**, 431–437 (2013). doi:10.1038/nature12352.
8. C. T. Brown, L. A. Hug, B. C. Thomas, I. Sharon, C. J. Castelle, A. Singh, M. J. Wilkins, K. C. Wrighton, K. H. Williams, and J. F. Banfield, "Unusual biology across a group comprising more than 15% of domain Bacteria," Nature. **523**, 208–211 (2015). doi:10.1038/nature14486.
9. L. A. Hug, B. J. Baker, K. Anantharaman, C. T. Brown, A. J. Probst, C. J. Castelle, C. N. Butterfield, A. W. Hernsdorf, Y. Amano, K. Ise *et al.*, "A new view of the tree of life," Nat Microbiol **1**, 16048 (2016). doi:10.1038/nmicrobiol.2016.48.
10. C. Quince, A. W. Walker, J. T. Simpson, N. J. Loman, and N. Segata, "Shotgun metagenomics, from sampling to analysis," Nat. Biotechnol. **35**, 833–844 (2017). doi:10.1038/nbt.3935.
11. A. Sczyrba, P. Hofmann, P. Belmann, D. Koslicki, S. Janssen, J. Dröge, I. Gregor, S. Majda, J. Fiedler, E. Dahms *et al.*, "Critical Assessment of Metagenome Interpretation—a benchmark of metagenomics software," Nat. Methods **14**, 1063–1071 (2017). doi:10.1038/nmeth.4458.
12. D. H. Parks, C. Rinke, M. Chuvochina, P. A. Chaumeil, B. J. Woodcroft, P. N. Evans, P. Hugenholtz, and G. W. Tyson, "Recovery of nearly 8,000 metagenome-assembled genomes substantially expands the tree of life," Nat Microbiol **2**, 1533–1542 (2017). doi:10.1038/s41564-017-0012-7.
13. B. J. Tully, E. D. Graham, and J. F. Heidelberg, "The reconstruction of 2,631 draft metagenome-assembled genomes from the global oceans," Sci Data **5**, 170203 (2018). doi:10.1038/sdata.2017.203.
14. R. D. Stewart, M. D. Auffret, A. Warr, A. H. Wiser, M. O. Press, K. W. Langford, I. Liachko, T. J. Snelling, R. J. Dewhurst, A. W. Walker *et al.*, "Assembly of 913 microbial genomes from metagenomic sequencing of the cow rumen," Nat Commun **9**, 870 (2018). doi:10.1038/s41467-018-03317-6.
15. T. O. Delmont, C. Quince, A. Shaiber, O. C. Esen, S. T. Lee, M. S. Rappe, S. L. MacLellan, S. Lucker, and A. M. Eren, "Nitrogen-fixing populations of Planctomycetes and Proteobacteria are abundant in surface ocean metagenomes," Nat Microbiol (2018). doi:10.1038/s41564-018-0176-9.
16. E. Pasolli, F. Asnicar, S. Manara, M. Zolfo, N. Karcher, F. Armanini, F. Beghini, P. Manghi, A. Tett, P. Ghensi *et al.*, "Extensive Unexplored Human Microbiome Diversity Revealed by Over 150,000 Genomes from Metagenomes Spanning Age, Geography, and Lifestyle," Cell **176**, 649–662 (2019). doi:10.1016/j.cell.2019.01.001.
17. T. Seemann, "Prokka: rapid prokaryotic genome annotation," Bioinformatics **30**, 2068–2069 (2014). doi:10.1093/bioinformatics/btu153.
18. B. J. Kunath, A. Bremges, A. Weimann, A. C. McHardy, and P. B. Pope, "Metagenomics and CAZyme Discovery," Methods Mol. Biol. **1588**, 255–277 (2017). doi:10.1007/978-1-4939-6899-2_20.
19. R. Feldbauer, F. Schulz, M. Horn, and T. Rattei, "Prediction of microbial phenotypes based on comparative genomics," BMC Bioinforma. **16 Suppl 14**, S1 (2015). doi:10.1186/1471-2105-16-S14-S1.
20. A. Weimann, K. Mooren, J. Frank, P. B. Pope, A. Bremges, and A. C. McHardy, "From Genomes to Phenotypes: Traitar, the Microbial Trait Analyzer," mSystems. **1** (2016). doi:10.1128/mSystems.00101-16.
21. R. Rosselló-Mora and R. Amann, "The species concept for prokaryotes," FEMS Microbiol. Rev. **25**, 39–67 (2001). doi:10.1111/j.1574-6976.2001.tb00571.x.
22. K. T. Konstantinidis and J. M. Tiedje, "Genomic insights that advance the species definition for prokaryotes," Proc. Natl. Acad. Sci. U.S.A. **102**, 2567–2572 (2005). doi:10.1073/pnas.0409727102.
23. P. Yarza, P. Yilmaz, E. Pruesse, F. O. Glockner, W. Ludwig, K. H. Schleifer, W. B. Whitman, J. Euzeby, R. Amann, and R. Rossello-Mora, "Uniting the classification of cultured and uncultured bacteria and archaea using 16S rRNA gene sequences," Nat. Rev. Microbiol. **12**, 635–645 (2014). doi:10.1038/nrmicro3330.
24. N. J. Varghese, S. Mukherjee, N. Ivanova, K. T. Konstantinidis, K. Mavrommatis, N. C. Kyrpides, and A. Pati, "Microbial species delineation using whole genome sequences," Nucleic Acids Res. **43**, 6761–6771 (2015). doi:10.1093/nar/gkv657.
25. D. H. Parks, M. Imelfort, C. T. Skennerton, P. Hugenholtz, and G. W. Tyson, "CheckM: assessing the quality of microbial genomes recovered from isolates, single cells, and metagenomes," Genome Res. **25**, 1043–1055 (2015). doi:10.1101/gr.186072.114.
26. R. D. Stewart, M. Auffret, T. J. Snelling, R. Roehe, and M. Watson, "MAGpy: a reproducible pipeline for the downstream analysis of metagenome-assembled genomes (MAGs)," Bioinformatics. (2018). doi:10.1093/bioinformatics/bty905.
27. A. M. Eren, O. C. Esen, C. Quince, J. H. Vineis, H. G. Morrison, M. L. Sogin, and T. O. Delmont, "Anvi'o: an advanced analysis and visualization platform for 'omics data," PeerJ. **3**, e1319 (2015). doi:10.7717/peerj.1319.
28. D. H. Huson, B. Albrecht, C. Bac, I. Bessarab, A. Górska, D. Jolic, and R. B. H. Williams, "MEGAN-LR: new algorithms allow accurate binning and easy interactive exploration of metagenomic long reads and contigs," Biol. Direct **13**, 6 (2018). doi:10.1186/s13062-018-0208-7.
29. C. C. Thompson, L. Chimetto, R. A. Edwards, J. Swings, E. Stackebrandt, and F. L. Thompson, "Microbial genomic taxonomy," BMC Genomics **14**, 913 (2013). doi:10.1186/1471-2164-14-913.
30. C. Jain, L. M. Rodriguez-R, A. M. Phillippy, K. T. Konstantinidis, and S. Aluru, "High throughput ANI analysis of 90K prokaryotic genomes reveals clear species boundaries," Nat Commun **9**, 5114 (2018). doi:10.1038/s41467-018-07641-9.
31. B. D. Ondov, T. J. Treangen, P. Melsted, A. B. Mallonee, N. H. Bergman, S. Koren, and A. M. Phillippy, "Mash: fast genome and metagenome distance estimation using MinHash," Genome Biol. **17**, 132 (2016). doi:10.1186/s13059-016-0997-x.
32. N. A. O'Leary, M. W. Wright, J. R. Brister, S. Ciufo, D. Haddad, R. McVeigh, B. Rajput, B. Robbertse, B. Smith-White, D. Ako-Adjei *et al.*, "Reference sequence (RefSeq) database at NCBI: current status, taxonomic expansion, and functional annotation," Nucleic Acids Res. **44**, D733–745 (2016). doi:10.1093/nar/gkv1189.
33. M. R. Olm, C. T. Brown, B. Brooks, and J. F. Banfield, "dRep: a tool for fast and accurate genomic comparisons that enables improved genome recovery from metagenomes through de-replication," ISME J **11**, 2864–2868 (2017). doi:10.1038/ismej.2017.126.
34. J. Pollock, L. Glendinning, T. Wisedchanwet, and M. Watson, "The madness of microbiome: Attempting to find consensus "Best Practice" for 16S microbiome studies," Appl. Environ. Microbiol. **84** (2018). doi:10.1128/AEM.02627-17.
35. R. Knight, A. Vrbanac, B. C. Taylor, A. Aksenov, C. Callewaert, J. Debelius, A. Gonzalez, T. Kosciolek, L. I. McCall, D. McDonald *et al.*, "Best practices for analysing microbiomes," Nat. Rev. Microbiol. **16**, 410–422 (2018). doi:10.1038/s41579-018-0029-9.
36. T. J. Wheeler and S. R. Eddy, "nhmmer: DNA homology search with profile HMMs," Bioinformatics. **29**, 2487–2489 (2013). doi:10.1093/bioinformatics/btt403.
37. B. J. Callahan, P. J. McMurdie, M. J. Rosen, A. W. Han, A. J. Johnson, and S. P. Holmes, "DADA2: High-resolution sample inference from Illumina amplicon data," Nat. Methods **13**, 581–583 (2016). doi:10.1038/nmeth.3869.
38. Q. Wang, G. M. Garrity, J. M. Tiedje, and J. R. Cole, "Naïve Bayesian classifier for rapid assignment of rRNA sequences into the new bacterial taxonomy," Appl. Environ. Microbiol. **73**, 5261–5267 (2007). doi:10.1128/AEM.00062-07.
39. C. Quast, E. Pruesse, P. Yilmaz, J. Gerken, T. Schweer, P. Yarza, J. Peplies, and F. O. Glöckner, "The SILVA ribso-

mal RNA gene database project: improved data processing and web-based tools," Nucleic Acids Res. **41**, D590–596 (2013). doi:10.1093/nar/gks1219.

40. J. R. Cole, Q. Wang, J. A. Fish, B. Chai, D. M. McGarrell, Y. Sun, C. T. Brown, A. Porras-Alfaro, C. R. Kuske, and J. M. Tiedje, "Ribosomal Database Project: data and tools for high throughput rRNA analysis," Nucleic Acids Res. **42**, D633–642 (2014). doi:10.1093/nar/gkt1244.
41. M. Balvoit and D. H. Huson, "SILVA, RDP, Greengenes, NCBI and OTT—how do these taxonomies compare?" BMC Genomics **18**, 114 (2017). doi:10.1186/s12864-017-3501-4.
42. S. Clingenpeel, A. Clum, P. Schwientek, C. Rinke, and T. Woyke, "Reconstructing each cell's genome within complex microbial communities-dream or reality?" Front Microbiol **5**, 771 (2014). doi:10.3389/fmicb.2014.00771.
43. A. Bremges, E. Singer, T. Woyke, and A. Sczyrba, "MeCorS: Metagenome-enabled error correction of single cell sequencing reads," Bioinformatics **32**, 2199–2201 (2016). doi:10.1093/bioinformatics/btw144.
44. P. Hugenholtz, A. Skarshewski, and D. H. Parks, "Genome-based microbial taxonomy coming of age," Cold Spring Harb Perspect Biol **8** (2016). doi:10.1101/cshperspect.a018085.
45. R. M. Bowers, N. C. Kyrpides, R. Stepanauskas, M. Harmon-Smith, D. Doud, T. B. K. Reddy, F. Schulz, J. Jarett, A. R. Rivers, E. A. Eloe-Fadrosh *et al.*, "Minimum information about a single amplified genome (MISAG) and a metagenome-assembled genome (MIMAG) of bacteria and archaea," Nat. Biotechnol. **35**, 725–731 (2017). doi:10.1038/nbt.3893.
46. T. Woyke, D. Tighe, K. Mavromatis, A. Clum, A. Copeland, W. Schackwitz, A. Lapidus, D. Wu, J. P. McCutcheon, B. R. McDonald *et al.*, "One bacterial cell, one complete genome," PLoS ONE **5**, e10314 (2010). doi:10.1371/journal.pone.0010314.
47. S. Krause, A. Bremges, P. C. Munch, A. C. McHardy, and J. Gescher, "Characterisation of a stable laboratory coculture of acidophilic nanoorganisms," Sci Rep **7**, 3289 (2017). doi:10.1038/s41598-017-03315-6.
48. D. Hyatt, G. L. Chen, P. F. Locascio, M. L. Land, F. W. Larimer, and L. J. Hauser, "Prodigal: prokaryotic gene recognition and translation initiation site identification," BMC Bioinforma. **11**, 119 (2010). doi:10.1186/1471-2105-11-119.
49. D. Kim, L. Song, F. P. Breitwieser, and S. L. Salzberg, "Centrifuge: rapid and sensitive classification of metagenomic sequences," Genome Res. **26**, 1721–1729 (2016). doi:10.1101/gr.210641.116.
50. P. Menzel, K. L. Ng, and A. Krogh, "Fast and sensitive taxonomic classification for metagenomics with Kaiju," Nat Commun **7**, 11257 (2016). doi:10.1038/ncomms11257.
51. E. W. Sayers, R. Agarwala, E. E. Bolton, J. R. Brister, K. Canese, K. Clark, R. Connor, N. Fiorini, K. Funk, T. Hefferon *et al.*, "Database resources of the National Center for Biotechnology Information," Nucleic Acids Res. **47**, D23–D28 (2019). doi:10.1093/nar/gky1069.
52. D. R. Mende, S. Sunagawa, G. Zeller, and P. Bork, "Accurate and universal delineation of prokaryotic species," Nat. Methods **10**, 881–884 (2013). doi:10.1038/nmeth.2575.
53. D. R. Mende, I. Letunic, J. Huerta-Cepas, S. S. Li, K. Forslund, S. Sunagawa, and P. Bork, "proGenomes: a resource for consistent functional and taxonomic annotations of prokaryotic genomes," Nucleic Acids Res. **45**, D529–D534 (2017). doi:10.1093/nar/gkw989.
54. F. A. Matsen, R. B. Kodner, and E. V. Armbrust, "pplacer: linear time maximum-likelihood and Bayesian phylogenetic placement of sequences onto a fixed reference tree," BMC Bioinforma. **11**, 538 (2010). doi:10.1186/1471-2105-11-538.
55. L. Czech, P. Barbera, and A. Stamatakis, "Methods for Automatic Reference Trees and Multilevel Phylogenetic Placement," Bioinformatics. (2018). doi:10.1093/bioinformatics/bty767.
56. P. Di Tommaso, M. Chatzou, E. W. Floden, P. P. Barja, E. Palumbo, and C. Notredame, "Nextflow enables reproducible computational workflows," Nat. Biotechnol. **35**, 316–319 (2017). doi:10.1038/nbt.3820.
57. A. Bremges, I. Maus, P. Belmann, F. Eikmeyer, A. Winkler, A. Albersmeier, A. Pühler, A. Schlüter, and A. Sczyrba, "Deeply sequenced metagenome and metatranscriptome of a biogas-producing microbial community from an agricultural production-scale biogas plant," GigaScience. **4**, 33 (2015). doi:10.1186/s13742-015-0073-6.
58. P. Belmann, J. Dröge, A. Bremges, A. C. McHardy, A. Sczyrba, and M. D. Barton, "Bioboxes: standardised containers for interchangeable bioinformatics software," GigaScience. **4**, 47 (2015). doi:10.1186/s13742-015-0087-0.
59. F. da Veiga Leprevost, B. A. Grüning, S. Alves Aflitos, H. L. Röst, J. Uszkoreit, H. Barsnes, M. Vaudel, P. Moreno, L. Gatto, J. Weber *et al.*, "BioContainers: an open-source and community-driven framework for software standardization," Bioinformatics. **33**, 2580–2582 (2017). doi:10.1093/bioinformatics/btx192.
60. B. Grüning, R. Dale, A. Sjödin, B. A. Chapman, J. Rowe, C. H. Tomkins-Tinch, R. Valieris, and J. Köster, "Bioconda: sustainable and comprehensive software distribution for the life sciences," Nat. Methods **15**, 475–476 (2018). doi:10.1038/s41592-018-0046-7.
61. S. Sunagawa, L. P. Coelho, S. Chaffron, J. R. Kultima, K. Labadie, G. Salazar, B. Djahanschiri, G. Zeller, D. R. Mende, A. Alberti *et al.*, "Ocean plankton. Structure and function of the global ocean microbiome," Science **348**, 1261359 (2015). doi:10.1126/science.1261359.
62. R. K. Aziz, D. Bartels, A. A. Best, M. DeJongh, T. Disz, R. A. Edwards, K. Formsma, S. Gerdes, E. M. Glass, M. Kubal *et al.*, "The RAST Server: rapid annotations using subsystems technology," BMC Genomics **9**, 75 (2008). doi:10.1186/1471-2164-9-75.
63. F. Meyer, P. Hofmann, P. Belmann, R. Garrido-Oter, A. Fritz, A. Sczyrba, and A. C. McHardy, "AMBER: Assessment of Metagenome BinnERs," Gigascience **7** (2018). doi:10.1093/gigascience/giy069.
64. A. Bremges and A. C. McHardy, "Critical Assessment of Metagenome Interpretation Enters the Second Round," mSystems **3** (2018). doi:10.1128/mSystems.00103-18.
65. A. Fritz, P. Hofmann, S. Majda, E. Dahms, J. Droge, J. Fiedler, T. R. Lesker, P. Belmann, M. Z. DeMaere, A. E. Darling *et al.*, "CAMISIM: simulating metagenomes and microbial communities," Microbiome **7**, 17 (2019). doi:10.1186/s40168-019-0633-6.
66. D. H. Parks, M. Chuvochina, D. W. Waite, C. Rinke, A. Skarshewski, P. A. Chaumeil, and P. Hugenholtz, "A standardized bacterial taxonomy based on genome phylogeny substantially revises the tree of life," Nat. Biotechnol. **36**, 996–1004 (2018). doi:10.1038/nbt.4229.
67. S. Federhen, "The NCBI Taxonomy database," Nucleic Acids Res. **40**, D136–143 (2012). doi:10.1093/nar/gkr1178.
