# Supplementary material for: CAMITAX: Taxon labels for microbial genomes
Source: Gigascience. 2020 Jan 7;9(1):giz154. doi: 10.1093/gigascience/giz154 (PMC6946028; doi:10.1093/gigascience/giz154)
Supplement: giz154_GIGA-D-19-00212_Revision_1 [file giz154_giga-d-19-00212_revision_1.pdf]

GigaScience

CAMITAX: Taxon labels for microbial genomes

--Manuscript Draft--

**Manuscript Number:**	GIGA-D-19-00212R1
**Full Title:**	CAMITAX: Taxon labels for microbial genomes
**Article Type:**	Technical Note
**Funding Information:**	
**Abstract:**	The number of microbial genome sequences is growing exponentially, also thanks to recent advances in recovering complete or near-complete genomes from metagenomes and single cells. Assigning reliable taxon labels to genomes is key and often a prerequisite for downstream analyses. We introduce CAMITAX, a scalable and reproducible workflow for the taxonomic labelling of microbial genomes recovered from isolates, single cells, and metagenomes. CAMITAX combines genome distance-, 16S rRNA gene-, and gene homology-based taxonomic assignments with phylogenetic placement. It uses Nextflow to orchestrate reference databases and software containers, and thus combines ease of installation and use with computational reproducibility. We evaluated the method on several hundred metagenome-assembled genomes with high-quality taxonomic annotations from the TARA Oceans project, and show that the ensemble classification method in CAMITAX improved on all individual methods across tested ranks. While we initially developed CAMITAX to aid the Critical Assessment of Metagenome Interpretation (CAMI) initiative, it evolved into a comprehensive software to reliably assign taxon labels to microbial genomes. CAMITAX is available under the Apache License 2.0 at: https://github.com/CAMI-challenge/CAMITAX
**Corresponding Author:**	Andreas Bremges<br><br>GERMANY
**Corresponding Author Secondary Information:**	
**Corresponding Author's Institution:**	
**Corresponding Author's Secondary Institution:**	
**First Author:**	Andreas Bremges
**First Author Secondary Information:**	
**Order of Authors:**	Andreas Bremges
	Adrian Fritz
	Alice C McHardy
**Order of Authors Secondary Information:**	
**Response to Reviewers:**	Thanks for the fair and streamlined review process so far. We also thank the reviewers for their constructive comments, which helped to improve the software, its documentation on GitHub, and - of course - the manuscript itself.<br><br>We highlighted any changes in the revised manuscript in bold and red. Please find our response to the minor points raised below; we believe to have addressed all of them and look forward to moving this forward!<br><br>> Reviewer #1: Bremges et al present an algorithm for assigning taxonomy to genomes. The manuscript is overall well written and the methods presented are sensible. I have some minor concerns about the current version of the manuscript, detailed below.

Powered by Editorial Manager® and ProduXion Manager® from Aries Systems Corporation

We thank the reviewer for his assessment and constructive comments.

> Abstract line 1: The word "also" should be removed.

We decided to leave the sentence as is. While culture-independent methods probably contribute the lion's share of new genome sequences, high-throughput cultivation techniques also enabled the reconstruction of thousands of new genome sequences (see e.g. Forster et al., Nature 2019; Zou et al., Nat Biotechnol 2019).

> 16S rRNA gene-based assignment. It isn't clear from the methods how the method deals with 16S genes which are incorrect i.e. misbinned or missassembled.

This is a valid and important point, thanks for raising it. We added the following to the classification algorithm section: "The trade-off is that incorrect individual assignments, e.g. due to potentially misassembled or misbinned 16S rRNA gene sequences in MAGs, can result in overly conservative assignments on high taxonomic ranks. CAMITAX therefore also reports the maximal root-to-leaf path as an alternative, and we suggest that the user investigates taxonomic discrepancies manually, taking individual assignments into account."

> Phylogenetic placement. The CheckM-based method is not especially well suited to the classification problem. I would not agree that CheckM is "state of the art" as a method for phylogeny-based taxonomy assignment, given the abundance of genomes and metagenome assembled genomes that have become available after CheckM was developed. It is perhaps conservative, as the authors suggest, but this may be more of a consequence of the age of the software. Ideally, the authors might develop or adapt some software for using a more up to date genome set for phylogenetic placement, but this shouldn't be a barrier to publication.

One can argue about CheckM being "state-of-the-art" but it is, without doubt, often used for exactly this purpose (also in software such as MAGpy; Stewart et al., Bioinformatics 2018). We believe that it still serves as a good-enough starting point for taxonomic analyses (as shown in Delmont et al., Nat Microbiol 2018) and thank the reviewer for the concluding remark. Silver lining: As far as we know, the CheckM authors are working on a revised version of their genome set and phylogenetic backbone tree (within the GTDB project), and possibly an CheckM successor, which we could then incorporate into CAMITAX.

> It would be preferable if the software would be written in Python 3 rather than Python 2, given the lack of official support for Python 2 beyond the end of this year. Given the small size of the software, an update would presumably not be onerous given a stated goal of the software is reproducibility.

We agree and, in fact, CAMITAX is already written in Python 3. CheckM, on the other hand, is Python 2 but we have no plans to re-implement CheckM in Python 3. Instead, to address the reviewer's point, we execute CheckM in a containerized environment (Docker or Singularity), fostering reproducibility and decoupling software (and Python version) dependencies from CAMITAX itself.

> I found Figure 2 somewhat unclear. Specifically, it wasn't immediately clear what the links represent, as it was not explained in the legend. Also, given that the Delmont et al set was used as the gold standard, then it would be more interpretable if the links emanated from there, rather than the CAMITAX column.

We apologize for the missing explanation and revised the legend, explaining the meaning of the links. It now reads: "Colored links between these ranks represent the "flow", i.e. changes in the assignment depth, between the three methods." The order was decided upon the Delmond et al. workflow: They started with the CheckM assignments (left) and then manually refined all assignments (middle). We contrast

Powered by Editorial Manager® and ProduXion Manager® from Aries Systems Corporation

their expert-curation with CAMITAX's fully automated workflow (right). We tried changing the order but in fact this made the figure much harder to read and interpret, and we therefore decided to leave it as is.

> In Figure 3, the meaning of the terms should be more fully explained. Specifically, it was not immediately clear how truncated taxonomies were treated compared to misclassifications.

We slightly revised the legend and now state how the performance metrics were computed: "Performance across ranks was assessed with the AMBER software using the manually assigned taxonomy by Delmont et al. as the gold standard." A full explanation, including specifics about handling truncated taxonomies, is probably too technical and we therefore refer to the AMBER manuscript (Meyer et al., GigaScience 2018) and documentation: https://github.com/CAMI-challenge/AMBER

> My understanding is that CheckM taxonomy is derived from IMG taxonomy, not NCBI. I'm not 100% sure that there is any difference between the two, but does the CAMITAX database gathering procedure check to make sure that the taxonomies produced from each tool are consistent with the "master" NCBI taxonomy?

It is correct that CheckM does not use the NCBI taxonomy and thus does not directly report NCBI taxonomy IDs for use in CAMITAX. It does, however, report the taxonomic lineage (with scientific names) and it has been shown that mapping by scientific name works reasonable well onto larger databases, such as NCBI (Balvociute & Huson, BMC Genomics 2017). We face a similar "problem" with the Dada2 results, by the way, where we use SILVA or RDP taxonomies, but - in either case - can map the reported scientific names to NCBI taxonomy IDs, worst-case with a slight loss in resolution, and eventually work on an internally consistent taxonomy. We found these implementation details to technical to be included in the manuscript.

> The installation procedure suggests perhaps building the databases from scratch, or using the nextflow tool to download the databases. Building from scratch is problematic because some of the download methods (e.g. the download from the NCBI FTP) procure files that are not versioned. Therefore 2 users building the database from scratch months apart will generate different reference databases, making CAMITAX not reproducible. A warning could be added to that section to advise users of this.

We 100% agree, thanks for pointing out this caveat. We added the following warning to the CAMITAX GitHub page: "Warning: To foster reproducibility, we strongly recommend that you use our "official" releases and we will continue to provide stable and versioned updates in the future."

> While CAMITAX purports to be reproducible, what this really means is that the build of the software is reproducible. It does not mean that 2 users running the same CAMITAX version with the same input data will end up with the same result, because of random number generators used within the software. For instance, running CheckM twice on the same input data can give divergent results because of randomness used in the tree insertion process. The authors should make this clear.

Fair enough and definitely worth pointing out, we added the following warning: "Warning: While CAMITAX is built around computational reproducibility, results might sometimes be slightly different from run to run (but of comparable quality) because software used within (e.g. CheckM) are non-deterministic."

> Reviewer #2: The paper by Bremges et al. describe CAMITAX a workflow designed for the taxonomic classification of microbial genomes obtained from the application of NGS-based methodologies, such as single-cell sequencing and metagenomics. Even if the 4 implemented methodologies itself do not represent a real novelty in the field, their harmonization by using a classification algorithm is interesting. Moreover, the idea to

Powered by Editorial Manager® and ProduXion Manager® from Aries Systems Corporation

deploy the workflow in a container greatly simplify both the installation and usage and ensure the analysis reproducibility.

We thank the reviewer for these kind words (and we obviously agree).

> The manuscript is well written and easy to read. All the proposed figures are appropriate and adequately support the data described in the main text. Figure 2 may be improved by using different colors allowing to easily discriminate the paths through the plot.

Thanks a lot. We found that working with a continuous color scheme worked out best for the alluvial diagram. While it is not perfect - and color choice always involves personal preferences -, the "viridis" color scheme captures the hierarchical ordering of the taxonomic ranks (with lighter colors representing higher ranks) and is perceptually uniform, robust to colorblindness, and prints reasonably well in grey scale.

> The CAMITAX GitHub repository clearly describe how to access and configure the container but very few information are available about the manual installation. The usage section needs an improvement.

We revised the CAMITAX GitHub page and added instructions how to install and run the software. Over the last months and by user feedback, however, we found that manual installation is very challenging (due to incompatible Python versions and external dependencies) and we recommend running the containerized version of CAMITAX. We improved native support for different compute environments (e.g. Singularity no longer requires manually fiddling with configuration files) and extended the documentation on GitHub: https://github.com/CAMI-challenge/CAMITAX

> I have some minor concerns about the paper:
> the classification algorithm needs to be described more in deep. A figure may help the readers;

Maybe the reviewer suspected something more "sophisticated" as our classification algorithm but CAMITAX selects the "most specific, yet consistent taxonomic label among all tools". Based on feedback from peers we provide three specific examples (using E. coli, a widely used organism for such toy examples) to illustrate the decision making and we don't think a generic figure can illustrate this step any better.

> regarding the overall drop of CAMITAX recall in mid-range ranks, I was wondering if it may be due to the fact that CAMITAX seems to be more conservative than the Delmont classification (figure 2). Authors should discuss in how many cases CAMITAX results more conservative than the reference classification.

We agree with the reviewer's comment (and note that this was implicitly captured by Fig. 2, as one can read the kingdom, phylum, class, order, family, genus, and species boxes as stacked bar charts). We now explicitly inserted the reviewer's observation into the main text: "While the recall of CAMITAX dropped at the mid-range ranks, largely due to a more conservative assignment strategy compared to Delmont et al.'s expert curation, it recovered for genus level assignments." Thanks!

> - Moreover, the authors claim that "Notably, 95% of CAMITAX's predictions were consistent with Delmont et al., i.e. the two assignments were on the same taxonomic lineage and their LCA is either of the two." Does it mean the authors consider consistent a classification for which CAMITAX assigns to the kingdom rank while Dermont assigns to species? Please clarify

Exactly, and Fig. 2 shows the extend different classification depths at scale. Please note, however, that there are no cases where Delmont et al. assigns to Species but CAMITAX assigns to the Kingdom rank, and only very few Genus/Kingdom cases; and

	it is expected that manual curation by experts outperforms any automatic approach.<br><br>> It would be useful to add some information about the technical requirements such as consumed RAM and required CPU time.<br><br>In its current version, we recommend at least 8 CPU cores and 24 GB of memory to execute CAMITAX. Following the reviewer's suggestion, we added this information to the CAMITAX GitHub page: https://github.com/CAMI-challenge/CAMITAX (under "Requirements"). We note that the requirements are due to the execution of bundled software and used databases, and the CAMITAX classification algorithm is (computationally) cheap.<br><br>Thanks again,<br>Andreas Bremges & co-authors
**Additional Information:**	
**Question**	**Response**
Are you submitting this manuscript to a special series or article collection?	No
**Experimental design and statistics**<br><br>Full details of the experimental design and statistical methods used should be given in the Methods section, as detailed in our Minimum Standards Reporting Checklist. Information essential to interpreting the data presented should be made available in the figure legends.<br><br>Have you included all the information requested in your manuscript?	Yes
**Resources**<br><br>A description of all resources used, including antibodies, cell lines, animals and software tools, with enough information to allow them to be uniquely identified, should be included in the Methods section. Authors are strongly encouraged to cite Research Resource Identifiers (RRIDs) for antibodies, model organisms and tools, where possible.<br><br>Have you included the information requested as detailed in our Minimum Standards Reporting Checklist?	Yes

**Availability of data and materials**<br><br>All datasets and code on which the conclusions of the paper rely must be either included in your submission or deposited in publicly available repositories (where available and ethically appropriate), referencing such data using a unique identifier in the references and in the “Availability of Data and Materials” section of your manuscript.<br><br>Have you have met the above requirement as detailed in our Minimum Standards Reporting Checklist?	Yes

Powered by Editorial Manager® and ProduXion Manager® from Aries Systems Corporation

revised version
Click here to access/download;Manuscript;camitax.pdf
Click here to view linked References

CAMITAX: Taxon labels for microbial genomes

Andreas Bremges[1,2,*], **Adrian Fritz**[1], and **Alice C. McHardy**[1,*]

[1] *Computational Biology of Infection Research, Helmholtz Centre for Infection Research, 38124 Braunschweig, Germany*
[2] *German Center for Infection Research (DZIF), partner site Hannover-Braunschweig, 38124 Braunschweig, Germany*

[*] *Correspondence: andreas.bremges@helmholtz-hzi.de; alice.mchardy@helmholtz-hzi.de*

The number of microbial genome sequences is growing exponentially, also thanks to recent advances in recovering complete or near-complete genomes from metagenomes and single cells. Assigning reliable taxon labels to genomes is key and often a prerequisite for downstream analyses. We introduce CAMITAX, a scalable and reproducible workflow for the taxonomic labelling of microbial genomes recovered from isolates, single cells, and metagenomes. CAMITAX combines genome distance-, 16S rRNA gene-, and gene homology-based taxonomic assignments with phylogenetic placement. It uses Nextflow to orchestrate reference databases and software containers, and thus combines ease of installation and use with computational reproducibility. We evaluated the method on several hundred metagenome-assembled genomes with high-quality taxonomic annotations from the TARA Oceans project, and show that the ensemble classification method in CAMITAX improved on all individual methods across tested ranks. While we initially developed CAMITAX to aid the Critical Assessment of Metagenome Interpretation (CAMI) initiative, it evolved into a comprehensive software to reliably assign taxon labels to microbial genomes. CAMITAX is available under the Apache License 2.0 at: https://github.com/CAMI-challenge/CAMITAX

INTRODUCTION

The direct costs for sequencing a microbial genome are at an all-time low: a high-quality draft now costs less than $100, a “finished” genome sequence less than $500. This resulted in many culture-dependent genome studies, in which thousands of isolates—selected by e.g. their distinct phylogeny [1, 2], abundance in the human microbiome [3, 4], or biotechnological relevance [5, 6]—are sequenced.

Single cell genome and shotgun metagenome studies further contribute to this expansion in genome numbers by enabling access to the genome sequences of (as-yet) uncultured microbes [7–9]. Notably, new bioinformatics methods can reconstruct complete or near-complete genomes even from complex environments [10, 11], and easily scale to hundreds or even thousands of metagenome samples [12–16].

Typically, the sequencing and assembly of a new genome is merely a prerequisite for further bioinformatics analyses (and their experimental validation) to uncover novel biological insights by e.g. functional annotation [17, 18] or phenotype prediction [19, 20], which often require the genome’s taxonomy.

Historically, a bacterial or archaeal species was defined as a collection of strains that share one (or more) trait(s) and show DNA-DNA reassociation values of 70% or higher [21]. However, with the advent of genomics and—more recently—culture-independent methods, this definition was found to be impractical and difficult to implement [22].

Today, 16S rRNA gene similarity, average nucleotide identity (ANI), genome phylogeny, or gene-centric voting schemes are used for taxonomic assignments [23–28]. These approaches all have their merits (see below), but, to the best of our knowledge, no unifying workflow implementation existed. To jointly use these complementary approaches, we developed CAMITAX, a scalable and reproducible workflow that combines genome distance-, 16S rRNA gene-, and gene homology-based taxonomic assignments with phylogenetic placement onto a fixed reference tree to reliably infer genome taxonomy.

METHODS

In the following, we describe CAMITAX’s assignment strategies and its implementation (Figure 1).

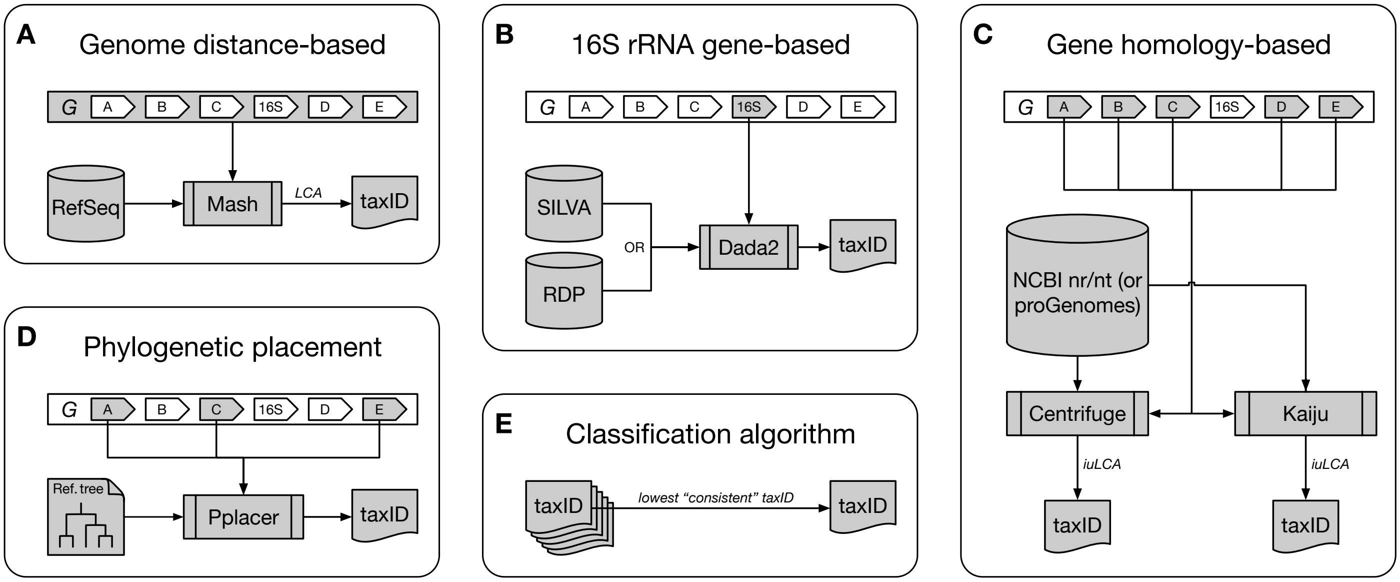

Fig. 1. The CAMITAX taxonomic assignment workflow. CAMITAX assigns one NCBI Taxonomy ID (taxID) to an input genome *G* by combining genome distance-, 16S rRNA gene-, and gene homology-based taxonomic assignments with phylogenetic placement. **(A) Genome distance-based assignment.** CAMITAX uses Mash to estimate the average nucleotide identity (ANI) between *G* and more than a hundred thousand microbial genomes in RefSeq, and assigns the lowest common ancestor (LCA) of genomes showing >95% ANI, which was found to be a clear species boundary. **(B) 16S rRNA gene-based assignment.** CAMITAX uses Dada2 to label *G*'s 16S rRNA gene sequences using the naïve Bayesian classifier method to assign taxonomy across multiple ranks (down to genus level), and exact sequence matching for species-level assignments, against the SILVA or RDP database. **(C) Gene homology-based assignments.** CAMITAX uses Centrifuge and Kaiju to perform gene homology searches against nucleotide and amino acid sequences in NCBI's nr and nt (or proGenomes' genes and proteins datasets), respectively. CAMITAX determines the interval-union LCA (iuLCA) of gene-level assignments and places *G* on the lowest taxonomic node with at least 50% coverage. **(D) Phylogenetic placement.** CAMITAX uses Pplacer to place *G* onto a fixed reference tree, as implemented in CheckM, and estimates genome completeness and contamination using lineage-specific marker genes. **(E) Classification algorithm.** CAMITAX considers the lowest consistent assignment as the longest unambiguous root-to-node path in the taxonomic tree spanned by the five taxIDs derived in (A)–(D), i.e. it retains the most specific, yet consistent taxonomic label among all tools.

Genome distance-based assignment

An ANI value of 95% roughly corresponds to a 70% DNA-DNA reassociation value (the historical species definition) [24]. In other words, strains from the same species are expected to show >95% ANI [29]. This species boundary appears to be widely applicable and has been confirmed in a recent large-scale study, in which the analyses of 8 billion genome pairs revealed a clear genetic discontinuity among known genomes, with 99.8% of the pairs showing either >95% intra-species ANI or <83% inter-species ANI values [30].

CAMITAX uses Mash [31] to rapidly estimate the input genomes' ANI to all bacterial or archaeal genomes in the RefSeq database [32] (114,176 strains as of 2018-05-10). CAMITAX's genome distance-based assignment is the lowest common ancestor (LCA) of all Mash hits with >95% ANI; a genome is placed at *root* if there is no RefSeq genome with >95% ANI.

This strategy works best if the query genome is more than 80% complete (Mash does not accurately estimate the genome-wide ANI of incomplete genomes [33]) and is represented in RefSeq. CAMITAX's other assignment strategies are complementary by design and better suited for incomplete genomes or underrepresented lineages. If a Mash hit is found, however, CAMITAX most likely assigns a taxonomy at the species or genus level.

16S rRNA gene-based assignment

The 16S rRNA gene is widely used for classification tasks because it is an universal marker gene likely present in all bacteria and archaea [34, 35].

CAMITAX uses nhmmer [36] to identify 16S rRNA genes in the input genomes and Dada2 [37] to assign taxonomy. Dada2 uses the naïve Bayesian classifier method [38] for kingdom to genus assignments, and exact sequence matching against a reference database for species assignments. CAMITAX supports two commonly used databases: SILVA [39] and RDP [40], which both were found to map back well to the NCBI Taxonomy [41].

Of course, this strategy only is applicable if the genome assembly contains a copy of the 16S rRNA gene—which is not always the case, particularly for genomes recovered from metagenomes or single cells.

Gene homology-based assignments

Metagenomics and single cell genomics are complementary approaches providing access to the genomes of (as-yet) uncultured microbes, but both have strings attached: Single amplified genomes (SAGs) suffer from amplification bias and, as a consequence, are often incomplete [42, 43]. Metagenome-assembled genomes (MAGs) on the other hand rarely contain full-length 16S rRNA genes [44, 45]. While there are notable exceptions to this rule [46, 47], the above assignment strategies are generally not expected to work well for today's SAGs and MAGs.

To overcome these problems, CAMITAX implements a gene-based voting scheme. It uses Prodigal [48] to predict protein-coding genes, and then Centrifuge [49] and Kaiju [50] for gene homology searches on the nucleotide and protein level, respectively. Both tools scale to large reference databases, such as NCBI's nr/nt [51], but (by default) CAMITAX resorts to the (much smaller) proGenomes genes and proteins datasets [52, 53]. The proGenomes database was designed as a resource for consistent taxonomic annotations of bacteria and archaea.

Inferring genome taxonomy from a set of gene-level assignments is not trivial, and—inspired by procedures implemented in anvi'o [27] and dRep [33]—CAMITAX places the query genome on the lowest taxonomic node with at least 50% support in gene assignments (which corresponds to the interval-union LCA algorithm [28]) for nucleotide and protein searches.

Phylogenetic placement

CAMITAX uses CheckM [25] for a phylogeny-driven estimate of taxonomy. Relying on 43 phylogenetically informative marker genes (consisting primarily of ribosomal proteins and RNA polymerase domains), CheckM places the query genome onto a fixed reference tree with Pplacer [54] to infer taxonomy. We note that phylogenetic placement is often quite conservative and does not necessarily provide resolution at the species level [26, 55].

Lastly, CAMITAX reports the query genome's completeness and contamination as estimated by CheckM using its lineage-specific marker genes [25].

Classification algorithm

CAMITAX considers the lowest consistent assignment as the longest unambiguous root-to-node path in the taxonomic tree spanned by the individual assignments, i.e. it retains the most specific, yet consistent taxonomic label among all tools. For example, CAMITAX would determine as "consistent" assignments for the individual assignments (derived with the different assignment strategies) the following:

- 3× *E. coli*, 2× *Bacteria* $\mapsto$ *E. coli*
- 3× *E. coli*, 2× *E. albertii* $\mapsto$ *Escherichia*
- 3× *E. coli*, 2× *Archaea* $\mapsto$ Root

This strategy is more robust than computing the lowest common ancestor (LCA) of individual assignments because outliers, e.g. missing predictions of conservative methods, don't affect the overall assignment.

At the same time, requiring a consistent assignment is less error-prone than e.g. selecting the maximal root-to-leaf path, which would introduce many false-positive assignments especially on lower ranks.

The trade-off is that incorrect individual assignments, e.g. due to potentially misassembled or misbinned 16S rRNA gene sequences in MAGs, can result in overly conservative assignments on high taxonomic ranks. CAMITAX therefore also reports the maximal root-to-leaf path as an alternative, and we suggest that the user investigates taxonomic discrepancies manually, taking individual assignments into account.

Implementation

CAMITAX incorporates many state-of-the-art pieces of software, and automatically resolves all software and database dependencies with Nextflow [56] in a containerized environment (Table 1). This fosters reproducibility in bioinformatics research [57, 58], and we strongly suggest to run CAMITAX using BioContainers [59] (automated container builds for software in bioconda [60]). CAMITAX can be run on a local machine or in a distributed fashion.

Table 1. Software used in the CAMITAX workflow.

Software	Version	BioContainer
Centrifuge	1.0.3	centrifuge:1.0.3–py36pl5.22.0_2
CheckM	1.0.11	checkm-genome:1.0.11–0
Dada2	1.6.0	bioconductor-dada2:1.6.0–r3.4.1_0
Kaiju	1.6.2	kaiju:1.6.2–pl5.22.0_0
Mash	2.0	mash:2.0–gsl2.2_2
Nhmmer	3.1	–
Pplacer	1.1	–
Prodigal	2.6.3	prodigal:2.6.3–0

CAMITAX automatically resolves all software dependencies with Nextflow using BioContainers in a containerized environment. Nhmmer and Pplacer are bundled with CheckM.

RESULTS

We applied CAMITAX to real data not present in its databases, a recent collection of 885 bacterial and archaeal MAGs from Delmont *et al.* [15], who used state-of-the-art metagenomic assembly, binning, and curation strategies to create a non-redundant database of microbial population genomes from the TARA Oceans project [61].

Delmont *et al.* used CheckM for an initial taxonomic inference of the MAGs. Thereafter, they used Centrifuge

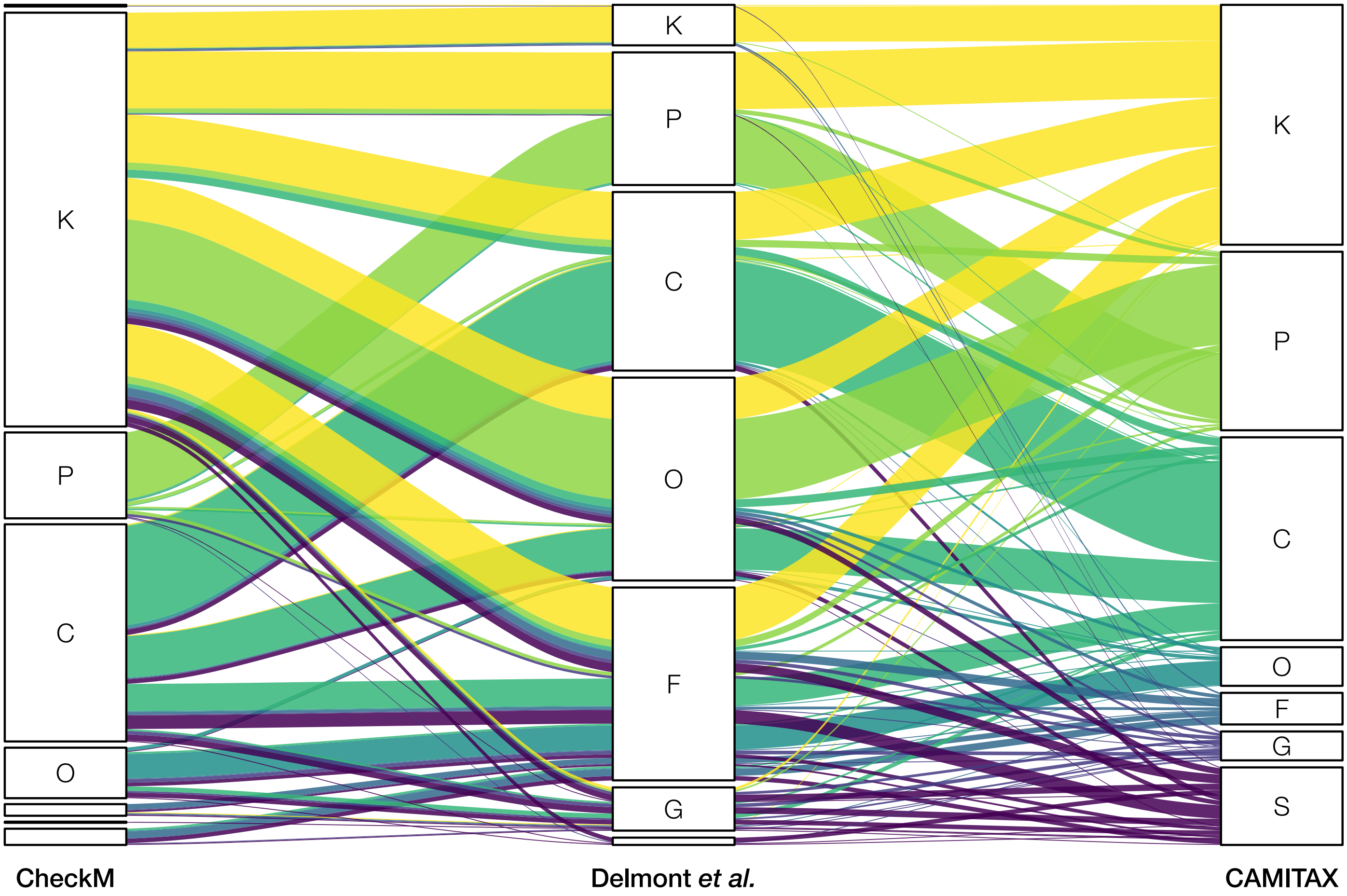

Fig. 2. Comparison of high quality taxonomic assignments for 885 MAGs. Using genome-resolved metagenomics, Delmont *et al.* assembled 885 bacterial and archaeal genomes from the TARA Oceans metagenomes and used CheckM for an initial taxonomic inference. Subsequently, they manually refined the taxonomic assignments using additional analyses and expert knowledge. The alluvial diagram shows the assigned taxonomic ranks for CheckM (left), manual curation (middle), and CAMITAX (right) on kingdom, phylum, class, order, family, genus, and species level. **Colored links between these ranks represent the "flow", i.e. changes in the assignment depth, between the three methods.**

[49], RAST [62], and manual BLAST searches of single-copy core genes against NCBI's nr/nt to manually refine their taxonomic inferences. Lastly, they trained a novel machine learning classifier to also identify MAGs affiliated to the Candidate Phyla Radiation (CPR) [8].

As expected, CAMITAX outperformed CheckM, which is rather conservative in its assignments, by adding low-ranking annotations based on high-quality predictions of other tools, such as Kaiju (Figure 2). Notably, 95% of CAMITAX's predictions were consistent with Delmont *et al.*, i.e. the two assignments were on the same taxonomic lineage and their LCA is either of the two. CAMITAX assignments of 46 MAGs (5%) were in conflict with the manually curated taxonomy. Of these, CAMITAX made species assignments for twelve MAGs based on Mash hits against RefSeq genomes. These we consider trustworthy because >95% ANI was shown to be a clear species boundary [30], and we assume that Delmont *et al.* assigned them incorrectly. On the other hand, CAMITAX for instance misclassified MAGs affiliated to the Candidate Phyla Radiation based on their 16S rRNA gene sequences to other phyla.

To quantify taxonomic assignment performance, we calculated precision, recall, and accuracy across all ranks with AMBER 2.0 [63] (Figure 3). As the gold standard, we used the Delmont *et al.* assignments up to genus rank. CAMITAX was very precise down to class level and reasonably (>80%) precise below. Overall, it was more accurate across all ranks than each of its assignment strategies individually. While the recall of CAMITAX dropped at the mid-range ranks, **largely due to a more conservative assignment strategy compared to Delmont *et al.*'s expert curation,** it recovered for genus level assignments.

We thus propose CAMITAX as a reliable and reproducible taxonomic assignment workflow, ideally followed by a manual refinement step—as always.

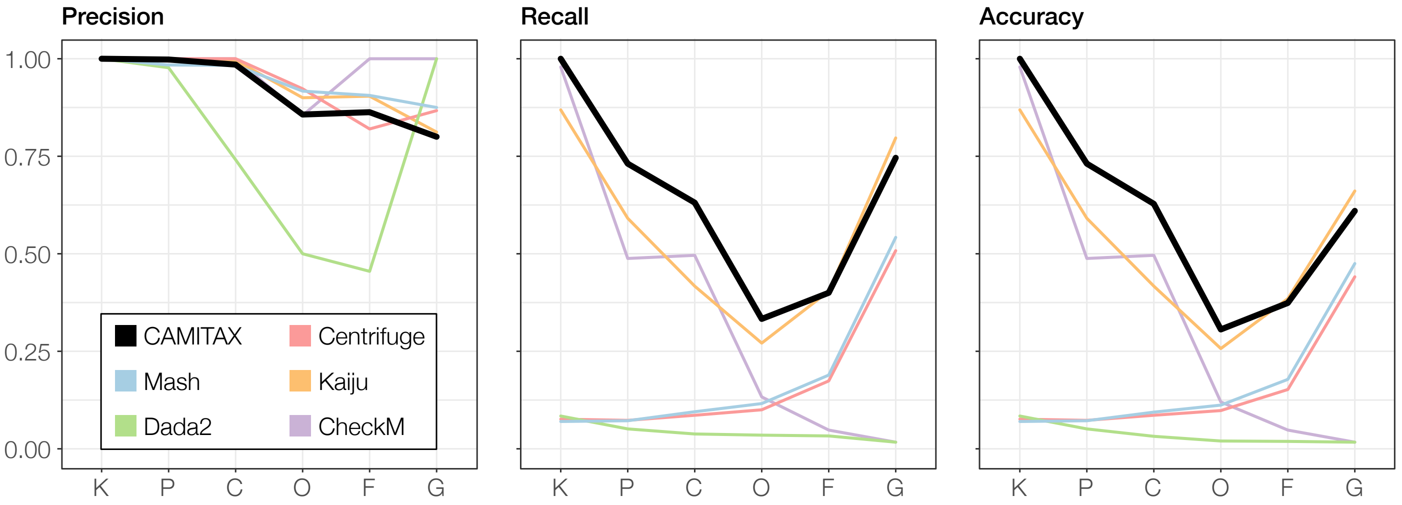

Fig. 3. Taxonomic assignment performance metrics across ranks for 885 MAGs. Performance across ranks was assessed with the AMBER software using the manually assigned taxonomy by Delmont *et al.* as the gold standard. Shown are precision, recall, and accuracy for CAMITAX (and the individual tools combined therein) on kingdom, phylum, class, order, family, and genus level.

DISCUSSION

CAMITAX was initially developed while preparing the second Critical Assessment of Metagenome Interpretation (CAMI) challenge [64]. The challenge datasets include new genomes from taxa (at different evolutionary distances) not found in public databases yet, which need high quality taxon labels for the subsequent microbial community and metagenome data simulation [65]. Due to this need, we created CAMITAX to systematically double-check, newly infer, or refine genome taxon label assignments in a fully reproducible way.

CAMITAX combines different taxonomic assignment strategies in one unifying workflow implementation. It uses Nextflow to orchestrate reference databases and software containers. Therefore, both databases and software can be easily substituted, providing the flexibility to cope with rapid change of standards oftentimes observed in the field. For instance, Parks *et al.* recently proposed a standardized bacterial taxonomy based on genome phylogeny, the so-called Genome Taxonomy Database (GTDB) [66]. While CAMITAX currently uses the NCBI Taxonomy [67], it is (at least in principle) agnostic to the underlying database and could thus be easily adapted to other taxonomy versions that will arise in future.

SOFTWARE AND DATA AVAILABILITY

CAMITAX is implemented in Nextflow and Python 3, and is freely available under the Apache License 2.0 at https://github.com/CAMI-challenge/CAMITAX.

Mash sketches for all bacterial and archaeal genomes in RefSeq, snapshots of the NCBI Taxonomy databases, and Centrifuge and Kaiju indices for the proGenomes genes and proteins datasets, respectively, are collected under doi:10.5281/zenodo.1250043. The snapshots used in this study, generated on 2018-05-10, are available under doi:10.5281/zenodo.1250044.

Dada2-formatted training fasta files, derived from SILVA (release 132) and RDP (training set 16, release 11.5), are available under doi:10.5281/zenodo.1172782 and doi:10.5281/zenodo.801827, respectively.

Lastly, the CheckM reference databases are available at https://data.ace.uq.edu.au/public/CheckM_databases.

AUTHORS' CONTRIBUTIONS

AB implemented the software, performed experiments, and wrote the paper with comments from AF and ACM. AF thoroughly tested the software. AB and ACM jointly conceived the project and evaluated results. All authors read and approved the final manuscript.

ACKNOWLEDGEMENTS

The authors thank Peter Belmann for Nextflow and Docker tips, Fernando Meyer for early beta testing, and the Isaac Newton Institute for Mathematical Sciences for its hospitality during the programme MTG, which was supported by EPSRC Grant Number EP/K032208/1.

REFERENCES

1. D. Wu, P. Hugenholtz, K. Mavromatis, R. Pukall, E. Dalin, N. N. Ivanova, V. Kunin, L. Goodwin, M. Wu, B. J. Tindall *et al.*, "A phylogeny-driven genomic encyclopaedia of Bacteria and Archaea," Nature **462**, 1056–1060 (2009). doi:10.1038/nature08656.
2. S. Mukherjee, R. Seshadri, N. J. Varghese, E. A. Eloe-Fadrosh, J. P. Meier-Kolthoff, M. Göker, R. C. Coates, M. Hadjithomas, G. A. Pavlopoulos, D. Paez-Espino *et al.*, "1,003 reference genomes of bacterial and archaeal isolates expand coverage of the tree of life," Nat. Biotechnol. **35**, 676–683 (2017). doi:10.1038/nbt.3886.
3. H. P. Browne, S. C. Forster, B. O. Anonye, N. Kumar, B. A. Neville, M. D. Stares, D. Goulding, and T. D. Lawley, "Culturing of 'uncul-

turable' human microbiota reveals novel taxa and extensive sporulation," Nature. **533**, 543–546 (2016). doi:10.1038/nature17645.
4. J. C. Lagier, S. Khelaifia, M. T. Alou, S. Ndongo, N. Dione, P. Hugon, A. Caputo, F. Cadoret, S. I. Traore, E. H. Seck *et al.*, "Culture of previously uncultured members of the human gut microbiota by culturomics," Nat Microbiol **1**, 16203 (2016). doi:10.1038/nmicrobiol.2016.203.
5. I. Maus, A. Bremges, Y. Stolze, S. Hahnke, K. G. Cibis, D. E. Koeck, Y. S. Kim, J. Kreubel, J. Hassa, D. Wibberg *et al.*, "Genomics and prevalence of bacterial and archaeal isolates from biogas-producing microbiomes," Biotechnol Biofuels **10**, 264 (2017). doi:10.1186/s13068-017-0947-1.
6. R. Seshadri, S. C. Leahy, G. T. Attwood, K. H. Teh, S. C. Lambie, A. L. Cookson, E. A. Eloe-Fadrosh, G. A. Pavlopoulos, M. Hadjithomas, N. J. Varghese *et al.*, "Cultivation and sequencing of rumen microbiome members from the Hungate1000 Collection," Nat. Biotechnol. **36**, 359–367 (2018). doi:10.1038/nbt.4110.
7. C. Rinke, P. Schwientek, A. Sczyrba, N. N. Ivanova, I. J. Anderson, J. F. Cheng, A. Darling, S. Malfatti, B. K. Swan, E. A. Gies *et al.*, "Insights into the phylogeny and coding potential of microbial dark matter," Nature. **499**, 431–437 (2013). doi:10.1038/nature12352.
8. C. T. Brown, L. A. Hug, B. C. Thomas, I. Sharon, C. J. Castelle, A. Singh, M. J. Wilkins, K. C. Wrighton, K. H. Williams, and J. F. Banfield, "Unusual biology across a group comprising more than 15% of domain Bacteria," Nature. **523**, 208–211 (2015). doi:10.1038/nature14486.
9. L. A. Hug, B. J. Baker, K. Anantharaman, C. T. Brown, A. J. Probst, C. J. Castelle, C. N. Butterfield, A. W. Hernsdorf, Y. Amano, K. Ise *et al.*, "A new view of the tree of life," Nat Microbiol **1**, 16048 (2016). doi:10.1038/nmicrobiol.2016.48.
10. C. Quince, A. W. Walker, J. T. Simpson, N. J. Loman, and N. Segata, "Shotgun metagenomics, from sampling to analysis," Nat. Biotechnol. **35**, 833–844 (2017). doi:10.1038/nbt.3935.
11. A. Sczyrba, P. Hofmann, P. Belmann, D. Koslicki, S. Janssen, J. Dröge, I. Gregor, S. Majda, J. Fiedler, E. Dahms *et al.*, "Critical Assessment of Metagenome Interpretation—a benchmark of metagenomics software," Nat. Methods **14**, 1063–1071 (2017). doi:10.1038/nmeth.4458.
12. D. H. Parks, C. Rinke, M. Chuvochina, P. A. Chaumeil, B. J. Woodcroft, P. N. Evans, P. Hugenholtz, and G. W. Tyson, "Recovery of nearly 8,000 metagenome-assembled genomes substantially expands the tree of life," Nat Microbiol **2**, 1533–1542 (2017). doi:10.1038/s41564-017-0012-7.
13. B. J. Tully, E. D. Graham, and J. F. Heidelberg, "The reconstruction of 2,631 draft metagenome-assembled genomes from the global oceans," Sci Data **5**, 170203 (2018). doi:10.1038/sdata.2017.203.
14. R. D. Stewart, M. D. Auffret, A. Warr, A. H. Wiser, M. O. Press, K. W. Langford, I. Liachko, T. J. Snelling, R. J. Dewhurst, A. W. Walker *et al.*, "Assembly of 913 microbial genomes from metagenomic sequencing of the cow rumen," Nat Commun **9**, 870 (2018). doi:10.1038/s41467-018-03317-6.
15. T. O. Delmont, C. Quince, A. Shaiber, O. C. Esen, S. T. Lee, M. S. Rappe, S. L. MacLellan, S. Lucker, and A. M. Eren, "Nitrogen-fixing populations of Planctomycetes and Proteobacteria are abundant in surface ocean metagenomes," Nat Microbiol (2018). doi:10.1038/s41564-018-0176-9.
16. E. Pasolli, F. Asnicar, S. Manara, M. Zolfo, N. Karcher, F. Armanini, F. Beghini, P. Manghi, A. Tett, P. Ghensi *et al.*, "Extensive Unexplored Human Microbiome Diversity Revealed by Over 150,000 Genomes from Metagenomes Spanning Age, Geography, and Lifestyle," Cell **176**, 649–662 (2019). doi:10.1016/j.cell.2019.01.001.
17. T. Seemann, "Prokka: rapid prokaryotic genome annotation," Bioinformatics **30**, 2068–2069 (2014). doi:10.1093/bioinformatics/btu153.
18. B. J. Kunath, A. Bremges, A. Weimann, A. C. McHardy, and P. B. Pope, "Metagenomics and CAZyme Discovery," Methods Mol. Biol. **1588**, 255–277 (2017). doi:10.1007/978-1-4939-6899-2_20.
19. R. Feldbauer, F. Schulz, M. Horn, and T. Rattei, "Prediction of microbial phenotypes based on comparative genomics," BMC Bioinforma. **16 Suppl 14**, S1 (2015). doi:10.1186/1471-2105-16-S14-S1.
20. A. Weimann, K. Mooren, J. Frank, P. B. Pope, A. Bremges, and A. C. McHardy, "From Genomes to Phenotypes: Traitar, the Microbial Trait Analyzer," mSystems. **1** (2016). doi:10.1128/mSystems.00101-16.
21. R. Rosselló-Mora and R. Amann, "The species concept for prokaryotes," FEMS Microbiol. Rev. **25**, 39–67 (2001). doi:10.1111/j.1574-6976.2001.tb00571.x.
22. K. T. Konstantinidis and J. M. Tiedje, "Genomic insights that advance the species definition for prokaryotes," Proc. Natl. Acad. Sci. U.S.A. **102**, 2567–2572 (2005). doi:10.1073/pnas.0409727102.
23. P. Yarza, P. Yilmaz, E. Pruesse, F. O. Glockner, W. Ludwig, K. H. Schleifer, W. B. Whitman, J. Euzeby, R. Amann, and R. Rossello-Mora, "Uniting the classification of cultured and uncultured bacteria and archaea using 16S rRNA gene sequences," Nat. Rev. Microbiol. **12**, 635–645 (2014). doi:10.1038/nrmicro3330.
24. N. J. Varghese, S. Mukherjee, N. Ivanova, K. T. Konstantinidis, K. Mavrommatis, N. C. Kyrpides, and A. Pati, "Microbial species delineation using whole genome sequences," Nucleic Acids Res. **43**, 6761–6771 (2015). doi:10.1093/nar/gkv657.
25. D. H. Parks, M. Imelfort, C. T. Skennerton, P. Hugenholtz, and G. W. Tyson, "CheckM: assessing the quality of microbial genomes recovered from isolates, single cells, and metagenomes," Genome Res. **25**, 1043–1055 (2015). doi:10.1101/gr.186072.114.
26. R. D. Stewart, M. Auffret, T. J. Snelling, R. Roehe, and M. Watson, "MAGpy: a reproducible pipeline for the downstream analysis of metagenome-assembled genomes (MAGs)," Bioinformatics. (2018). doi:10.1093/bioinformatics/bty905.
27. A. M. Eren, O. C. Esen, C. Quince, J. H. Vineis, H. G. Morrison, M. L. Sogin, and T. O. Delmont, "Anvi'o: an advanced analysis and visualization platform for 'omics data," PeerJ. **3**, e1319 (2015). doi:10.7717/peerj.1319.
28. D. H. Huson, B. Albrecht, C. Bağcı, I. Bessarab, A. Górska, D. Jolic, and R. B. H. Williams, "MEGAN-LR: new algorithms allow accurate binning and easy interactive exploration of metagenomic long reads and contigs," Biol. Direct **13**, 6 (2018). doi:10.1186/s13062-018-0208-7.
29. C. C. Thompson, L. Chimetto, R. A. Edwards, J. Swings, E. Stackebrandt, and F. L. Thompson, "Microbial genomic taxonomy," BMC Genomics **14**, 913 (2013). doi:10.1186/1471-2164-14-913.
30. C. Jain, L. M. Rodriguez-R, A. M. Phillippy, K. T. Konstantinidis, and S. Aluru, "High throughput ANI analysis of 90K prokaryotic genomes reveals clear species boundaries," Nat Commun **9**, 5114 (2018). doi:10.1038/s41467-018-07641-9.
31. B. D. Ondov, T. J. Treangen, P. Melsted, A. B. Mallonee, N. H. Bergman, S. Koren, and A. M. Phillippy, "Mash: fast genome and metagenome distance estimation using MinHash," Genome Biol. **17**, 132 (2016). doi:10.1186/s13059-016-0997-x.
32. N. A. O'Leary, M. W. Wright, J. R. Brister, S. Ciufo, D. Haddad, R. McVeigh, B. Rajput, B. Robbertse, B. Smith-White, D. Ako-Adjei *et al.*, "Reference sequence (RefSeq) database at NCBI: current status, taxonomic expansion, and functional annotation," Nucleic Acids Res. **44**, D733–745 (2016). doi:10.1093/nar/gkv1189.
33. M. R. Olm, C. T. Brown, B. Brooks, and J. F. Banfield, "dRep: a tool for fast and accurate genomic comparisons that enables improved genome recovery from metagenomes through de-replication," ISME J **11**, 2864–2868 (2017). doi:10.1038/ismej.2017.126.
34. J. Pollock, L. Glendinning, T. Wisedchanwet, and M. Watson, "The madness of microbiome: Attempting to find consensus "Best Practice" for 16S microbiome studies," Appl. Environ. Microbiol. **84** (2018). doi:10.1128/AEM.02627-17.
35. R. Knight, A. Vrbanac, B. C. Taylor, A. Aksenov, C. Callewaert, J. Debelius, A. Gonzalez, T. Kosciolek, L. I. McCall, D. McDonald *et al.*, "Best practices for analysing microbiomes," Nat. Rev. Microbiol. **16**, 410–422 (2018). doi:10.1038/s41579-018-0029-9.
36. T. J. Wheeler and S. R. Eddy, "nhmmer: DNA homology search with profile HMMs," Bioinformatics. **29**, 2487–2489 (2013). doi:10.1093/bioinformatics/btt403.
37. B. J. Callahan, P. J. McMurdie, M. J. Rosen, A. W. Han, A. J. Johnson, and S. P. Holmes, "DADA2: High-resolution sample inference from Illumina amplicon data," Nat. Methods **13**, 581–583 (2016). doi:10.1038/nmeth.3869.
38. Q. Wang, G. M. Garrity, J. M. Tiedje, and J. R. Cole, "Naïve Bayesian classifier for rapid assignment of rRNA sequences into

the new bacterial taxonomy," Appl. Environ. Microbiol. **73**, 5261–5267 (2007). doi:10.1128/AEM.00062-07.

39. C. Quast, E. Pruesse, P. Yilmaz, J. Gerken, T. Schweer, P. Yarza, J. Peplies, and F. O. Glöckner, "The SILVA ribosomal RNA gene database project: improved data processing and web-based tools," Nucleic Acids Res. **41**, D590–596 (2013). doi:10.1093/nar/gks1219.
40. J. R. Cole, Q. Wang, J. A. Fish, B. Chai, D. M. McGarrell, Y. Sun, C. T. Brown, A. Porras-Alfaro, C. R. Kuske, and J. M. Tiedje, "Ribosomal Database Project: data and tools for high throughput rRNA analysis," Nucleic Acids Res. **42**, D633–642 (2014). doi:10.1093/nar/gkt1244.
41. M. Balvočiūtė and D. H. Huson, "SILVA, RDP, Greengenes, NCBI and OTT—how do these taxonomies compare?" BMC Genomics **18**, 114 (2017). doi:10.1186/s12864-017-3501-4.
42. S. Clingenpeel, A. Clum, P. Schwientek, C. Rinke, and T. Woyke, "Reconstructing each cell's genome within complex microbial communities-dream or reality?" Front Microbiol **5**, 771 (2014). doi:10.3389/fmicb.2014.00771.
43. A. Bremges, E. Singer, T. Woyke, and A. Sczyrba, "MeCorS: Metagenome-enabled error correction of single cell sequencing reads," Bioinformatics **32**, 2199–2201 (2016). doi:10.1093/bioinformatics/btw144.
44. P. Hugenholtz, A. Skarshewski, and D. H. Parks, "Genome-based microbial taxonomy coming of age," Cold Spring Harb Perspect Biol **8** (2016). doi:10.1101/cshperspect.a018085.
45. R. M. Bowers, N. C. Kyrpides, R. Stepanauskas, M. Harmon-Smith, D. Doud, T. B. K. Reddy, F. Schulz, J. Jarett, A. R. Rivers, E. A. Eloe-Fadrosh *et al.*, "Minimum information about a single amplified genome (MISAG) and a metagenome-assembled genome (MIMAG) of bacteria and archaea," Nat. Biotechnol. **35**, 725–731 (2017). doi:10.1038/nbt.3893.
46. T. Woyke, D. Tighe, K. Mavromatis, A. Clum, A. Copeland, W. Schackwitz, A. Lapidus, D. Wu, J. P. McCutcheon, B. R. McDonald *et al.*, "One bacterial cell, one complete genome," PLoS ONE **5**, e10314 (2010). doi:10.1371/journal.pone.0010314.
47. S. Krause, A. Bremges, P. C. Munch, A. C. McHardy, and J. Gescher, "Characterisation of a stable laboratory co-culture of acidophilic nanoorganisms," Sci Rep **7**, 3289 (2017). doi:10.1038/s41598-017-03315-6.
48. D. Hyatt, G. L. Chen, P. F. Locascio, M. L. Land, F. W. Larimer, and L. J. Hauser, "Prodigal: prokaryotic gene recognition and translation initiation site identification," BMC Bioinforma. **11**, 119 (2010). doi:10.1186/1471-2105-11-119.
49. D. Kim, L. Song, F. P. Breitwieser, and S. L. Salzberg, "Centrifuge: rapid and sensitive classification of metagenomic sequences," Genome Res. **26**, 1721–1729 (2016). doi:10.1101/gr.210641.116.
50. P. Menzel, K. L. Ng, and A. Krogh, "Fast and sensitive taxonomic classification for metagenomics with Kaiju," Nat Commun **7**, 11257 (2016). doi:10.1038/ncomms11257.
51. E. W. Sayers, R. Agarwala, E. E. Bolton, J. R. Brister, K. Canese, K. Clark, R. Connor, N. Fiorini, K. Funk, T. Hefferon *et al.*, "Database resources of the National Center for Biotechnology Information," Nucleic Acids Res. **47**, D23–D28 (2019). doi:10.1093/nar/gky1069.
52. D. R. Mende, S. Sunagawa, G. Zeller, and P. Bork, "Accurate and universal delineation of prokaryotic species," Nat. Methods **10**, 881–884 (2013). doi:10.1038/nmeth.2575.
53. D. R. Mende, I. Letunic, J. Huerta-Cepas, S. S. Li, K. Forslund, S. Sunagawa, and P. Bork, "proGenomes: a resource for consistent functional and taxonomic annotations of prokaryotic genomes," Nucleic Acids Res. **45**, D529–D534 (2017). doi:10.1093/nar/gkw989.
54. F. A. Matsen, R. B. Kodner, and E. V. Armbrust, "pplacer: linear time maximum-likelihood and Bayesian phylogenetic placement of sequences onto a fixed reference tree," BMC Bioinforma. **11**, 538 (2010). doi:10.1186/1471-2105-11-538.
55. L. Czech, P. Barbera, and A. Stamatakis, "Methods for Automatic Reference Trees and Multilevel Phylogenetic Placement," Bioinformatics. (2018). doi:10.1093/bioinformatics/bty767.
56. P. Di Tommaso, M. Chatzou, E. W. Floden, P. P. Barja, E. Palumbo, and C. Notredame, "Nextflow enables reproducible computational workflows," Nat. Biotechnol. **35**, 316–319 (2017). doi:10.1038/nbt.3820.
57. A. Bremges, I. Maus, P. Belmann, F. Eikmeyer, A. Winkler, A. Albersmeier, A. Pühler, A. Schlüter, and A. Sczyrba, "Deeply sequenced metagenome and metatranscriptome of a biogas-producing microbial community from an agricultural production-scale biogas plant," GigaScience. **4**, 33 (2015). doi:10.1186/s13742-015-0073-6.
58. P. Belmann, J. Dröge, A. Bremges, A. C. McHardy, A. Sczyrba, and M. D. Barton, "Bioboxes: standardised containers for interchangeable bioinformatics software," GigaScience. **4**, 47 (2015). doi:10.1186/s13742-015-0087-0.
59. F. da Veiga Leprevost, B. A. Grüning, S. Alves Aflitos, H. L. Röst, J. Uszkoreit, H. Barsnes, M. Vaudel, P. Moreno, L. Gatto, J. Weber *et al.*, "BioContainers: an open-source and community-driven framework for software standardization," Bioinformatics. **33**, 2580–2582 (2017). doi:10.1093/bioinformatics/btx192.
60. B. Grüning, R. Dale, A. Sjödin, B. A. Chapman, J. Rowe, C. H. Tomkins-Tinch, R. Valieris, and J. Köster, "Bioconda: sustainable and comprehensive software distribution for the life sciences," Nat. Methods **15**, 475–476 (2018). doi:10.1038/s41592-018-0046-7.
61. S. Sunagawa, L. P. Coelho, S. Chaffron, J. R. Kultima, K. Labadie, G. Salazar, B. Djahanschiri, G. Zeller, D. R. Mende, A. Alberti *et al.*, "Ocean plankton. Structure and function of the global ocean microbiome," Science **348**, 1261359 (2015). doi:10.1126/science.1261359.
62. R. K. Aziz, D. Bartels, A. A. Best, M. DeJongh, T. Disz, R. A. Edwards, K. Formsma, S. Gerdes, E. M. Glass, M. Kubal *et al.*, "The RAST Server: rapid annotations using subsystems technology," BMC Genomics **9**, 75 (2008). doi:10.1186/1471-2164-9-75.
63. F. Meyer, P. Hofmann, P. Belmann, R. Garrido-Oter, A. Fritz, A. Sczyrba, and A. C. McHardy, "AMBER: Assessment of Metagenome BinnERs," Gigascience **7** (2018). doi:10.1093/gigascience/giy069.
64. A. Bremges and A. C. McHardy, "Critical Assessment of Metagenome Interpretation Enters the Second Round," mSystems **3** (2018). doi:10.1128/mSystems.00103-18.
65. A. Fritz, P. Hofmann, S. Majda, E. Dahms, J. Droge, J. Fiedler, T. R. Lesker, P. Belmann, M. Z. DeMaere, A. E. Darling *et al.*, "CAMISIM: simulating metagenomes and microbial communities," Microbiome **7**, 17 (2019). doi:10.1186/s40168-019-0633-6.
66. D. H. Parks, M. Chuvochina, D. W. Waite, C. Rinke, A. Skarshewski, P. A. Chaumeil, and P. Hugenholtz, "A standardized bacterial taxonomy based on genome phylogeny substantially revises the tree of life," Nat. Biotechnol. **36**, 996–1004 (2018). doi:10.1038/nbt.4229.
67. S. Federhen, "The NCBI Taxonomy database," Nucleic Acids Res. **40**, D136–143 (2012). doi:10.1093/nar/gkr1178.
