# Supplementary material for: CAMITAX: Taxon labels for microbial genomes
Source: Gigascience. 2020 Jan 7;9(1):giz154. doi: 10.1093/gigascience/giz154 (PMC6946028; doi:10.1093/gigascience/giz154)
Supplement: giz154_Response_to_Reviewer_Comments_Original_Submission [file giz154_response_to_reviewer_comments_original_submission.pdf]

Author's Response To Reviewer Comments

Close

Thanks for the fair and streamlined review process so far. We also thank the reviewers for their constructive comments, which helped to improve the software, its documentation on GitHub, and - of course - the manuscript itself.

We highlighted any changes in the revised manuscript in bold and red. Please find our response to the minor points raised below; we believe to have addressed all of them and look forward to moving this forward!

> Reviewer #1: Bremges et al present an algorithm for assigning taxonomy to genomes. The manuscript is overall well written and the methods presented are sensible. I have some minor concerns about the current version of the manuscript, detailed below.

We thank the reviewer for his assessment and constructive comments.

> Abstract line 1: The word "also" should be removed.

We decided to leave the sentence as is. While culture-independent methods probably contribute the lion's share of new genome sequences, high-throughput cultivation techniques also enabled the reconstruction of thousands of new genome sequences (see e.g. Forster et al., Nature 2019; Zou et al., Nat Biotechnol 2019).

> 16S rRNA gene-based assignment. It isn't clear from the methods how the method deals with 16S genes which are incorrect i.e. misbinned or missassembled.

This is a valid and important point, thanks for raising it. We added the following to the classification algorithm section: "The trade-off is that incorrect individual assignments, e.g. due to potentially misassembled or misbinned 16S rRNA gene sequences in MAGs, can result in overly conservative assignments on high taxonomic ranks. CAMITAX therefore also reports the maximal root-to-leaf path as an alternative, and we suggest that the user investigates taxonomic discrepancies manually, taking individual assignments into account."

> Phylogenetic placement. The CheckM-based method is not especially well suited to the classification problem. I would not agree that CheckM is "state of the art" as a method for phylogeny-based taxonomy assignment, given the abundance of genomes and metagenome assembled genomes that have become available after CheckM was developed. It is perhaps conservative, as the authors suggest, but this may be more of a consequence of the age of the software. Ideally, the authors might develop or adapt some software for using a more up to date genome set for phylogenetic placement, but this shouldn't be a barrier to publication.

One can argue about CheckM being "state-of-the-art" but it is, without doubt, often used for exactly this purpose (also in software such as MAGpy; Stewart et al., Bioinformatics 2018). We believe that it still serves as a good-enough starting point for taxonomic analyses (as shown in Delmont et al., Nat Microbiol 2018) and thank the reviewer for the concluding remark. Silver lining: As far as we know, the CheckM authors are working on a revised version of their genome set and phylogenetic backbone tree (within the GTDB project), and possibly an CheckM successor, which we could then incorporate into CAMITAX.

> It would be preferable if the software would be written in Python 3 rather than Python 2, given the

lack of official support for Python 2 beyond the end of this year. Given the small size of the software, an update would presumably not be onerous given a stated goal of the software is reproducibility.

We agree and, in fact, CAMITAX is already written in Python 3. CheckM, on the other hand, is Python 2 but we have no plans to re-implement CheckM in Python 3. Instead, to address the reviewer's point, we execute CheckM in a containerized environment (Docker or Singularity), fostering reproducibility and decoupling software (and Python version) dependencies from CAMITAX itself.

> I found Figure 2 somewhat unclear. Specifically, it wasn't immediately clear what the links represent, as it was not explained in the legend. Also, given that the Delmont et al set was used as the gold standard, then it would be more interpretable if the links emanated from there, rather than the CAMITAX column.

We apologize for the missing explanation and revised the legend, explaining the meaning of the links. It now reads: "Colored links between these ranks represent the "flow", i.e. changes in the assignment depth, between the three methods." The order was decided upon the Delmond et al. workflow: They started with the CheckM assignments (left) and then manually refined all assignments (middle). We contrast their expert-curation with CAMITAX's fully automated workflow (right). We tried changing the order but in fact this made the figure much harder to read and interpret, and we therefore decided to leave it as is.

> In Figure 3, the meaning of the terms should be more fully explained. Specifically, it was not immediately clear how truncated taxonomies were treated compared to misclassifications.

We slightly revised the legend and now state how the performance metrics were computed: "Performance across ranks was assessed with the AMBER software using the manually assigned taxonomy by Delmont et al. as the gold standard." A full explanation, including specifics about handling truncated taxonomies, is probably too technical and we therefore refer to the AMBER manuscript (Meyer et al., GigaScience 2018) and documentation: https://github.com/CAMI-challenge/AMBER

> My understanding is that CheckM taxonomy is derived from IMG taxonomy, not NCBI. I'm not 100% sure that there is any difference between the two, but does the CAMITAX database gathering procedure check to make sure that the taxonomies produced from each tool are consistent with the "master" NCBI taxonomy?

It is correct that CheckM does not use the NCBI taxonomy and thus does not directly report NCBI taxonomy IDs for use in CAMITAX. It does, however, report the taxonomic lineage (with scientific names) and it has been shown that mapping by scientific name works reasonable well onto larger databases, such as NCBI (Balvociute & Huson, BMC Genomics 2017). We face a similar "problem" with the Dada2 results, by the way, where we use SILVA or RDP taxonomies, but - in either case - can map the reported scientific names to NCBI taxonomy IDs, worst-case with a slight loss in resolution, and eventually work on an internally consistent taxonomy. We found these implementation details to technical to be included in the manuscript.

> The installation procedure suggests perhaps building the databases from scratch, or using the nextflow tool to download the databases. Building from scratch is problematic because some of the download methods (e.g. the download from the NCBI FTP) procure files that are not versioned. Therefore 2 users building the database from scratch months apart will generate different reference databases, making CAMITAX not reproducible. A warning could be added to that section to advise users of this.

We 100% agree, thanks for pointing out this caveat. We added the following warning to the CAMITAX GitHub page: "Warning: To foster reproducibility, we strongly recommend that you use our "official" releases and we will continue to provide stable and versioned updates in the future."

> While CAMITAX purports to be reproducible, what this really means is that the build of the software is reproducible. It does not mean that 2 users running the same CAMITAX version with the same input

data will end up with the same result, because of random number generators used within the software. For instance, running CheckM twice on the same input data can give divergent results because of randomness used in the tree insertion process. The authors should make this clear.

Fair enough and definitely worth pointing out, we added the following warning: "Warning: While CAMITAX is built around computational reproducibility, results might sometimes be slightly different from run to run (but of comparable quality) because software used within (e.g. CheckM) are non-deterministic."

> Reviewer #2: The paper by Bremges et al. describe CAMITAX a workflow designed for the taxonomic classification of microbial genomes obtained from the application of NGS-based methodologies, such as single-cell sequencing and metagenomics. Even if the 4 implemented methodologies itself do not represent a real novelty in the field, their harmonization by using a classification algorithm is interesting. Moreover, the idea to deploy the workflow in a container greatly simplify both the installation and usage and ensure the analysis reproducibility.

We thank the reviewer for these kind words (and we obviously agree).

> The manuscript is well written and easy to read. All the proposed figures are appropriate and adequately support the data described in the main text. Figure 2 may be improved by using different colors allowing to easily discriminate the paths through the plot.

Thanks a lot. We found that working with a continuous color scheme worked out best for the alluvial diagram. While it is not perfect - and color choice always involves personal preferences -, the "viridis" color scheme captures the hierarchical ordering of the taxonomic ranks (with lighter colors representing higher ranks) and is perceptually uniform, robust to colorblindness, and prints reasonably well in grey scale.

> The CAMITAX GitHub repository clearly describe how to access and configure the container but very few information are available about the manual installation. The usage section needs an improvement.

We revised the CAMITAX GitHub page and added instructions how to install and run the software. Over the last months and by user feedback, however, we found that manual installation is very challenging (due to incompatible Python versions and external dependencies) and we recommend running the containerized version of CAMITAX. We improved native support for different compute environments (e.g. Singularity no longer requires manually fiddling with configuration files) and extended the documentation on GitHub: https://github.com/CAMI-challenge/CAMITAX

> I have some minor concerns about the paper:
> the classification algorithm needs to be described more in deep. A figure may help the readers;

Maybe the reviewer suspected something more "sophisticated" as our classification algorithm but CAMITAX selects the "most specific, yet consistent taxonomic label among all tools". Based on feedback from peers we provide three specific examples (using E. coli, a widely used organism for such toy examples) to illustrate the decision making and we don't think a generic figure can illustrate this step any better.

> regarding the overall drop of CAMITAX recall in mid-range ranks, I was wondering if it may be due to the fact that CAMITAX seems to be more conservative than the Delmont classification (figure 2). Authors should discuss in how many cases CAMITAX results more conservative than the reference classification.

We agree with the reviewer's comment (and note that this was implicitly captured by Fig. 2, as one can read the kingdom, phylum, class, order, family, genus, and species boxes as stacked bar charts). We now explicitly inserted the reviewer's observation into the main text: "While the recall of CAMITAX dropped at the mid-range ranks, largely due to a more conservative assignment strategy compared to Delmont et al.’s expert curation, it recovered for genus level assignments." Thanks!

> - Moreover, the authors claim that "Notably, 95% of CAMITAX's predictions were consistent with Delmont et al., i.e. the two assignments were on the same taxonomic lineage and their LCA is either of the two." Does it mean the authors consider consistent a classification for which CAMITAX assigns to the kingdom rank while Dermont assigns to species? Please clarify

Exactly, and Fig. 2 shows the extend different classification depths at scale. Please note, however, that there are no cases where Delmont et al. assigns to Species but CAMITAX assigns to the Kingdom rank, and only very few Genus/Kingdom cases; and it is expected that manual curation by experts outperforms any automatic approach.

> It would be useful to add some information about the technical requirements such as consumed RAM and required CPU time.

In its current version, we recommend at least 8 CPU cores and 24 GB of memory to execute CAMITAX. Following the reviewer's suggestion, we added this information to the CAMITAX GitHub page: https://github.com/CAMI-challenge/CAMITAX (under "Requirements"). We note that the requirements are due to the execution of bundled software and used databases, and the CAMITAX classification algorithm is (computationally) cheap.

Thanks again,
Andreas Bremges & co-authors

Close
