# Supplementary material for: CAMITAX: Taxon labels for microbial genomes
Source: Gigascience. 2020 Jan 7;9(1):giz154. doi: 10.1093/gigascience/giz154 (PMC6946028; doi:10.1093/gigascience/giz154)
Supplement: giz154_Response_to_Reviewer_Comments_Revision_1 [file giz154_response_to_reviewer_comments_revision_1.pdf]

Author's Response To Reviewer Comments

Close

Dear Nicole,

thanks for your assistance uploading the accepted version.
I have incorporated your requested changes (GigaDB DOI).

Best,
Andreas

Close
