# Supplementary material for: CAMITAX: Taxon labels for microbial genomes
Source: Gigascience. 2020 Jan 7;9(1):giz154. doi: 10.1093/gigascience/giz154 (PMC6946028; doi:10.1093/gigascience/giz154)
Supplement: giz154_Reviewer_1_Report_Original_Submission — Ben Woodcroft -- 7/29/2019 Reviewed [file giz154_reviewer_1_report_original_submission.pdf]

Reviewer Report

Title: CAMITAX: Taxon labels for microbial genomes

Version: Original Submission **Date:** 7/29/2019

Reviewer name: Ben Woodcroft

Reviewer Comments to Author:

Bremges et al present an algorithm for assigning taxonomy to genomes. The manuscript is overall well written and the methods presented are sensible. I have some minor concerns about the current version of the manuscript, detailed below.
Abstract line 1: The word "also" should be removed.
16S rRNA gene-based assignment. It isn't clear from the methods how the method deals with 16S genes which are incorrect i.e. misbinned or missassembled.
Phylogenetic placement. The CheckM-based method is not especially well suited to the classification problem. I would not agree that CheckM is "state of the art" as a method for phylogeny-based taxonomy assignment, given the abundance of genomes and metagenome assembled genomes that have become available after CheckM was developed. It is perhaps conservative, as the authors suggest, but this may be more of a consequence of the age of the software. Ideally, the authors might develop or adapt some software for using a more up to date genome set for phylogenetic placement, but this shouldn't be a barrier to publication.
It would be preferable if the software would be written in Python 3 rather than Python 2, given the lack of official support for Python 2 beyond the end of this year. Given the small size of the software, an update would presumably not be onerous given a stated goal of the software is reproducibility.
I found Figure 2 somewhat unclear. Specifically, it wasn't immediately clear what the links represent, as it was not explained in the legend. Also, given that the Delmont et al set was used as the gold standard, then it would be more interpretable if the links emanated from there, rather than the CAMITAX column.
In Figure 3, the meaning of the terms should be more fully explained. Specifically, it was not immediately clear how truncated taxonomies were treated compared to misclassifications.
My understanding is that CheckM taxonomy is derived from IMG taxonomy, not NCBI. I'm not 100% sure that there is any difference between the two, but does the CAMITAX database gathering procedure check to make sure that the taxonomies produced from each tool are consistent with the "master" NCBI taxonomy?
The installation procedure suggests perhaps building the databases from scratch, or using the nextflow tool to download the databases. Building from scratch is problematic because some of the download methods (e.g. the download from the NCBI FTP) procure files that are not versioned. Therefore 2 users building the database from scratch months apart will generate different reference databases, making CAMITAX not reproducible. A warning could be added to that section to advise users of this.
While CAMITAX purports to be reproducible, what this really means is that the build of the software is reproducible. It does not mean that 2 users running the same CAMITAX version with the same input data will end up with the same result, because of random number generators used within the software.

For instance, running CheckM twice on the same input data can give divergent results because of randomness used in the tree insertion process. The authors should make this clear.

Level of Interest

Please indicate how interesting you found the manuscript: Choose an item.

Quality of Written English

Please indicate the quality of language in the manuscript: Choose an item.

Declaration of Competing Interests

Please complete a declaration of competing interests, considering the following questions:

- Have you in the past five years received reimbursements, fees, funding, or salary from an organisation that may in any way gain or lose financially from the publication of this manuscript, either now or in the future?
- Do you hold any stocks or shares in an organisation that may in any way gain or lose financially from the publication of this manuscript, either now or in the future?
- Do you hold or are you currently applying for any patents relating to the content of the manuscript?
- Have you received reimbursements, fees, funding, or salary from an organization that holds or has applied for patents relating to the content of the manuscript?
- Do you have any other financial competing interests?
- Do you have any non-financial competing interests in relation to this paper?

If you can answer no to all of the above, write 'I declare that I have no competing interests' below. If your reply is yes to any, please give details below.

I declare that I have no competing interests

I agree to the open peer review policy of the journal. I understand that my name will be included on my report to the authors and, if the manuscript is accepted for publication, my named report including any attachments I upload will be posted on the website along with the authors' responses. I agree for my report to be made available under an Open Access Creative Commons CC-BY license (http://creativecommons.org/licenses/by/4.0/). I understand that any comments which I do not wish to be included in my named report can be included as confidential comments to the editors, which will not be published.

Choose an item.

To further support our reviewers, we have joined with Publons, where you can gain additional credit to further highlight your hard work (see: https://publons.com/journal/530/gigascience). On publication of this paper, your review will be automatically added to Publons, you can then choose whether or not to claim your Publons credit. I understand this statement.

Yes Choose an item.
