# Supplementary material for: CAMITAX: Taxon labels for microbial genomes
Source: Gigascience. 2020 Jan 7;9(1):giz154. doi: 10.1093/gigascience/giz154 (PMC6946028; doi:10.1093/gigascience/giz154)
Supplement: giz154_Reviewer_2_Report_Original_Submission — Bruno Fosso -- 8/6/2019 Reviewed [file giz154_reviewer_2_report_original_submission.pdf]

Reviewer Report

Title: CAMITAX: Taxon labels for microbial genomes

Version: Original Submission **Date:** 8/6/2019

Reviewer name: Bruno Fosso

Reviewer Comments to Author:

The paper by Bremges et al. describe CAMITAX a workflow designed for the taxonomic classification of microbial genomes obtained from the application of NGS-based methodologies, such as single-cell sequencing and metagenomics. Even if the 4 implemented methodologies itself do not represent a real novelty in the field, their harmonization by using a classification algorithm is interesting. Moreover, the idea to deploy the workflow in a container greatly simplify both the installation and usage and ensure the analysis reproducibility.

The manuscript is well written and easy to read. All the proposed figures are appropriate and adequately support the data described in the main text. Figure 2 may be improved by using different colors allowing to easily discriminate the paths through the plot.

The CAMITAX GitHub repository clearly describe how to access and configure the container but very few information are available about the manual installation. The usage section needs an improvement.

I have some minor concerns about the paper:

- the classification algorithm needs to be described more in deep. A figure may help the readers;
- regarding the overall drop of CAMITAX recall in mid-range ranks, I was wondering if it may be due to the fact that CAMITAX seems to be more conservative than the Delmont classification (figure 2). Authors should discuss in how many cases CAMITAX results more conservative than the reference classification.
- Moreover, the authors claim that "Notably, 95% of CAMITAX's predictions were consistent with Delmont et al., i.e. the two assignments were on the same taxonomic lineage and their LCA is either of the two." Does it mean the authors consider consistent a classification for which CAMITAX assigns to the kingdom rank while Dermont assigns to species? Please clarify

It would be useful to add some information about the technical requirements such as consumed RAM and required CPU time.

Level of Interest

Please indicate how interesting you found the manuscript: Choose an item.

Quality of Written English

Please indicate the quality of language in the manuscript: Choose an item.

Declaration of Competing Interests

Please complete a declaration of competing interests, considering the following questions:

- Have you in the past five years received reimbursements, fees, funding, or salary from an organisation that may in any way gain or lose financially from the publication of this manuscript, either now or in the future?
- Do you hold any stocks or shares in an organisation that may in any way gain or lose financially from the publication of this manuscript, either now or in the future?
- Do you hold or are you currently applying for any patents relating to the content of the manuscript?
- Have you received reimbursements, fees, funding, or salary from an organization that holds or has applied for patents relating to the content of the manuscript?
- Do you have any other financial competing interests?
- Do you have any non-financial competing interests in relation to this paper?

If you can answer no to all of the above, write 'I declare that I have no competing interests' below. If your reply is yes to any, please give details below.

I declare that I have no competing interests

I agree to the open peer review policy of the journal. I understand that my name will be included on my report to the authors and, if the manuscript is accepted for publication, my named report including any attachments I upload will be posted on the website along with the authors' responses. I agree for my report to be made available under an Open Access Creative Commons CC-BY license (http://creativecommons.org/licenses/by/4.0/). I understand that any comments which I do not wish to be included in my named report can be included as confidential comments to the editors, which will not be published.

Choose an item.

To further support our reviewers, we have joined with Publons, where you can gain additional credit to further highlight your hard work (see: https://publons.com/journal/530/gigascience). On publication of this paper, your review will be automatically added to Publons, you can then choose whether or not to claim your Publons credit. I understand this statement.

Yes Choose an item.
